# Active Learning‐Guided Accelerated Discovery of Ultra‐Efficient High‐Entropy Thermoelectrics

**DOI:** 10.1002/adma.202515054

**Published:** 2025-10-09

**Authors:** Hanhwi Jang, Wooseok Lee, Hwa‐Jung Kim, Sohyang Cha, Hosun Shin, Won Bo Lee, Min‐Wook Oh, Yeon Sik Jung, YongJoo Kim

**Affiliations:** ^1^ Department of Materials Science and Engineering Korea Advanced Institute of Science and Technology Daejeon 34141 Republic of Korea; ^2^ School of Chemical and Biological Engineering Seoul National University Seoul 08826 Republic of Korea; ^3^ Division of Chemical and Materials Metrology Korea Research Institute of Standards and Science (KRISS) Daejeon 34113 Republic of Korea; ^4^ School of Transdisciplinary Innovations Seoul National University Seoul 08826 Republic of Korea; ^5^ Department of Materials Science and Engineering Hanbat National University Daejeon 34158 Republic of Korea; ^6^ Department of Materials Science and Engineering Korea University Seoul 02841 Republic of Korea

**Keywords:** active learning, disordered, efficiency, high‐entropy chalcogenides, thermoelectrics

## Abstract

High‐entropy alloys are emerging as highly efficient thermoelectrics, but their vast compositional spaces hinder efficient material discovery using conventional heuristics‐based and advanced machine learning approaches. Here, this fundamental challenge is addressed by demonstrating an active learning framework that leverages sparse experimental data (80 out of 16206) to efficiently identify three new high‐entropy chalcogenides (HECs) with remarkable thermoelectric performance (*zT* >2). By integrating physics‐informed descriptors with uncertainty‐aware sampling, this model efficiently assimilates latent structure–property relationships. This allows for systematic exclusion of unfavorable chemistries, enabling even non‐experts in thermoelectrics to design unexplored systems with arbitrary components. Furthermore, novel atomic arrangements and distinctive electron and phonon transport properties are uncovered, which are responsible for the superior performance in HECs, advancing the understanding of physical phenomena in disorder‐rich systems.

## Graphical abstract

An active learning framework is introduced for the accelerated discovery of high‐entropy chalcogenides with superior thermoelectric performance. Only 80 targeted syntheses, selected from 16206 possible combinations, led to three high‐performance compositions, demonstrating the remarkable efficiency of data‐driven guidance in experimental materials design.

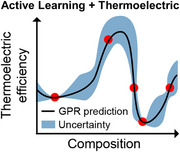

## 1 Introduction

Thermoelectric materials have garnered significant attention due to their ability to convert heat into electrical energy or vice versa.^[^ ^1^ ^]^ Given that over 70% of global energy consumption is dissipated as waste heat, utilizing thermoelectric generators to harvest electricity presents a promising approach to reducing reliance on conventional fossil fuels.^[^ ^2^, ^3^ ^]^ Furthermore, thermoelectric active cooling has been proposed as a viable alternative to passive thermal management techniques, addressing local hot spot formation in highly integrated semiconductors.^[^ ^4^ ^]^ Consequently, developing highly efficient thermoelectric materials is essential for advancing carbon neutrality and effectively regulating heat in modern electronic devices.

Despite their potential, improving thermoelectric conversion efficiency remains a complex challenge due to the intricate trade‐offs between transport properties.^[^ ^5^ ^]^ To tackle this, diverse materials such as chalcogenides,^[^ ^6^ ^]^ oxyselenides,^[^ ^7^ ^]^ Zintl compounds,^[^ ^8^ ^]^ half‐Heuslers,^[^ ^9^ ^]^ and skutterudites^[^ ^10^ ^]^ have been extensively studied over the past three decades in an effort to discover high‐performance thermoelectric materials. These materials are often doped or alloyed with external elements to enhance transport properties. However, two significant challenges arise in this experimental approach: i) the thermodynamic stability and corresponding doping solubility of guest elements may restrict the accessible design space,^[^ ^11^ ^]^ and ii) the dopant concentration or alloying fraction is often chosen arbitrarily, heavily relying on the domain knowledge of experts rather than well‐defined design principles.^[^ ^12^ ^]^

A promising strategy to overcome solubility limitations in multi‐elemental systems is the introduction of configurational disorder. The increased configurational entropy compensates for the enthalpic penalty associated with forming a solid solution, enabling the stabilization of a single‐phase alloy across a broad compositional space.^[^ ^13^ ^]^ This high‐entropy phase expands the design space, unlocking new opportunities to optimize thermoelectric properties beyond conventional alloy systems.^[^ ^14^, ^15^ ^]^ Notably, several efficient high‐entropy thermoelectric alloys have been experimentally realized, underscoring their potential as a promising class of thermoelectric materials.^[^ ^16^, ^17^, ^18^ ^]^ In addition to thermoelectrics, high‐entropy alloys exhibit promising functional properties such as superconductivity,^[^ ^19^, ^20^, ^21^ ^]^ mechanical robustness,^[^ ^22^, ^23^ ^]^ and catalytic activity,^[^ ^24^, ^25^ ^]^ underscoring their wide applicability.

However, optimizing the composition of constituent elements in high‐entropy materials introduces new challenges, making conventional trial‐and‐error approaches impractical, especially when experiments are costly and time‐consuming due to the large composition space.^[^ ^26^ ^]^ Computational methods such as density functional theory (DFT) calculations combined with machine learning techniques have been proposed to simulate properties of virtual alloys and predict high‐performance compositions prior to experimentation.^[^ ^27^, ^28^, ^29^, ^30^ ^]^ Despite these advancements, intrinsic limitations of DFT hinder the discovery process of high‐entropy thermoelectric materials: i) inaccuracies in describing the crystal structures of high‐entropy alloys with ionic or covalent bonds (e.g., oxides or chalcogenides),^[^ ^31^ ^]^ ii) difficulties in modeling electron and phonon relaxation times in disordered systems,^[^ ^32^ ^]^ and iii) the inability to account for unavoidable experimental noise in synthesis and measurements. As a result, compounds predicted to exhibit superior performance often fall short of expectations, even when machine learning models exhibit high accuracy.^[^ ^33^, ^34^ ^]^ Furthermore, the scarcity of extensive datasets on the thermoelectric properties of high‐entropy alloys exacerbates these issues, diminishing the ability of machine learning models to accurately predict transport properties (See **Table** **1**). To address these challenges, it is imperative to develop innovative methodologies that facilitate the efficient discovery of high‐entropy thermoelectric materials.

**Table 1:** Representative studies on machine learning–assisted thermoelectric discovery compared with this work.

Refs.	Material system	Dataset size	Machine learning method	Descriptor [s]	Validation	Key outcome	Limitation
[48]	Half‐Heuslers	≈79 000	Random forst, linear regression	Atomic radii, electronegativity, Pettifor scale	Computational only	Predicted 3 new half‐Heusler compounds with lattice thermal conductivity <5 W m^−1^ K^−1^	No experimental syshesis, thermal conductivity only
[49]	Zintl compounds	344	Gradient Boosting Regressor	Atomic radius, lattice constant, specific heat	Computation only	Identified 5 new candidates with lattice thermal conductivity < 2 W m^−1^ K^−1^	No experimental synthesis, thermal conductivity only
[50]	Cu‐doped Bi_2_Te_2.85_Se_0.15_ alloys	7	Artificial Neural Network with Bayesian Optimization	Cu content, density, texturing factor, grain size, etc.	No experimental validation: Only cross‐validation for machine learning	Predicted *zT* (max 0.86) consistent with experiment	Overfitting due to severe data scarcity, unphysical prediction
[51]	BiSbTe‐based composites	24	Bayesian Optimization with a Gaussian Process Regression	Binder concentration, extrusion pressure	Experimental validation: fabrication and measurement under the conditions predicted by the model	Achieving a *zT* of 1.3 at room temperature	Used for process optimization, not a material discovery
[52]	Multiple thermoelectric systems (Mg_3_Sb_2_, Bi_2_Te_3_, Zintl, half‐Heuslers, oxides)	≈3000	Conditional variational autoencoding generative adversarial networks (CVAEGAN)	65D composition vectors, *zT* using one‐hot encoding scheme	DFT for TiFe_2_Sn, ZrFeSe, and TiFeSe. Experimental Validation for Mg_3.1_Sb_0.5_Bi_1.497_Te_0.003_	Generated 100 doped candidates with *zT* >1.0, 25 novel compounds	Experimental validation limited to one well‐known family
This work	High‐entropy chalcogenides (9‐element, 16206 candidates)	80	Gaussian Process Regression with Bayesian Optimization	Thermoelectric quality factor (*B*)	Experimental validation: high‐performance candidates were synthesized and measured	Discovered 3 HECs with *zT*>2 by exploring only ≈0.5% of the design space	Averaging *B* over temperature to train the model

In this study, we demonstrate that active learning combined with targeted experiments enables the discovery of three high‐performance chalcogenides from a vast nonary compositional design space comprising 16206 candidates. Remarkably, the entire discovery process involved experimental synthesis and measurement of only 80 samples, accounting for less than 0.5% of the total design space. This exceptional screening capability is achieved by incorporating average quality factor (*B*_avg_) as a physics‐informed descriptor and employing a sophisticated balance between exploration and exploitation during the active learning process. Notably, the model iteratively assimilates latent knowledge on thermoelectric properties from experimental feedback, enabling it to exclude elements detrimental to overall performance. This suggests that even experimentalists inexperienced in thermoelectrics can randomly select elements for synthesis while relying on the active learning model to exclude unfavorable components. Finally, we retrospectively investigate the identified high‐performance compositions and extract the possible physical mechanisms underlying the observed performance improvements.

## 2 Overcoming Fragmented Material Discovery in Thermoelectrics

**Figure 1:**
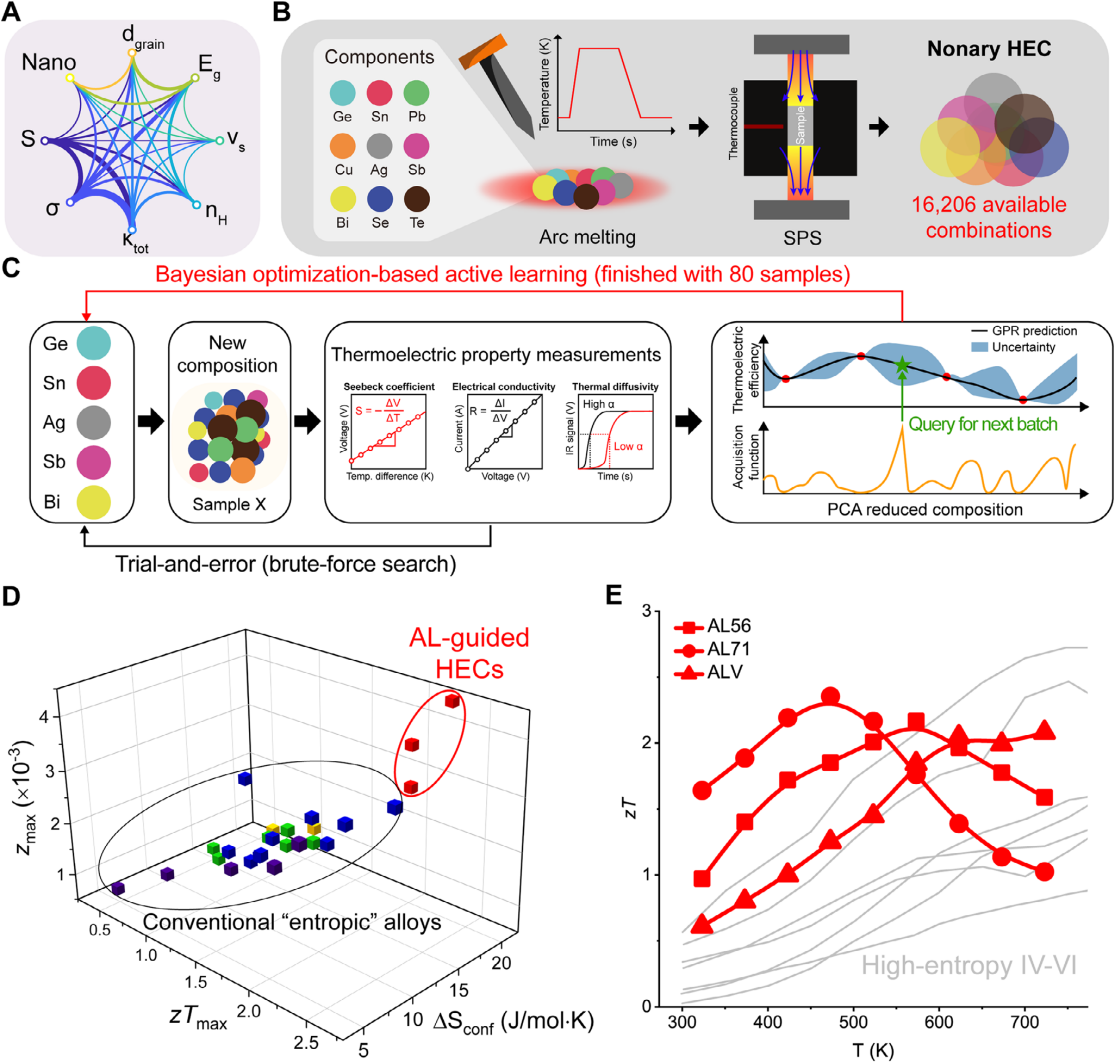
Design of high entropy chalcogenides (HECs) to overcome thermoelectric trade‐offs. A) Chord diagram illustrating the interrelationships between key thermoelectric parameters, with thicker connections representing stronger correlations. B) High‐throughput synthesis workflow for HECs consisting of nine elements, enabling melting, pulverization, and sintering within less than an hour. C) Closed‐loop experimental framework integrating conventional trial‐and‐error methods with Bayesian optimization‐based active learning, accelerating thermoelectric material discovery. D) Comparison of configurational entropy, maximum *zT* (*zT*_max_), and the maximum *z* (*z*_max_) of conventional high entropy thermoelectric alloys and the newly developed HECs. E) Temperature‐dependent *zT* values of previously reported high‐entropy IV‐VI thermoelectrics compared to the active learning‐guided HECs, demonstrating enhanced thermoelectric properties across a broad temperature range. Same color denotes the same sample in (D) and (E). References for (E) are provided in the Supporting Information.

The development of novel thermoelectric materials has been significantly hindered by strong trade‐offs among key thermoelectric parameters, as illustrated in **Figure** **1**A. Consequently, sequential optimization of these parameters is nearly impossible, necessitating a labor‐intensive trial‐and‐error approach guided by expert design heuristics.^[^ ^35^ ^]^ However, performance improvements in conventional thermoelectric materials have stagnated due to limited design capabilities and the absence of universal design principles governing thermoelectric materials.^[^ ^36^, ^37^ ^]^

This challenge becomes even more pronounced when developing entirely new thermoelectric materials. For example, aliovalent doping is a widely adopted strategy to manipulate the Seebeck coefficient and electrical conductivity, often accompanied by reduced thermal conductivity due to increased phonon scattering.^[^ ^38^ ^]^ While initial doping may enhance thermoelectric performance, further improvements are often limited, particularly when a second dopant modifies carrier concentration or induces bipolar thermal conduction, potentially offsetting the benefits introduced by the first dopant.^[^ ^39^, ^40^ ^]^ In such cases, the dopant selection process must be repeated iteratively, often with limited transferability of design principles, resulting in inefficient and fragmented material optimization efforts.

To overcome these limitations, we hypothesized that simultaneous multi‐element optimization under fixed processing conditions could mitigate the constraints of sequential optimization. This led us to develop high‐entropy chalcogenides (HECs) as a model system (Figure 1B). HECs offer an unprecedentedly large compositional space with extended solubility, making them ideal for co‐optimizing multiple elements simultaneously. The HECs in this study incorporate nine elements (Ge, Sn, Pb, Cu, Ag, Sb, Bi, Se, and Te), yielding 16206 possible compositions even with design constraints (see Supporting Methods for details). To efficiently explore this vast compositional space, we employed a high‐throughput thermoelectric material synthesis process using vacuum arc melting and spark plasma sintering (SPS).^[^ ^41^ ^]^ This process enables synthesis in less than an hour, a significant improvement over conventional melting and solidification methods, which typically require more than a day.^[^ ^42^ ^]^ However, despite this accelerated synthesis, synthesizing all possible compositions would still take 675 days, assuming sequential processing, necessitating a more efficient decision‐making and optimization strategy.

Traditionally, selecting the most promising compositions for further experimentation relies on the knowledge‐based expert intuition, where researchers make informed selections based on measured thermoelectric properties (Figure 1C).^[^ ^35^ ^]^ However, given the extreme compositional complexity of HECs and the intricate correlations between constituent elements, this approach becomes highly nuanced and impractical. These challenges can be mitigated by implementing Bayesian active learning, a machine learning framework that systematically and iteratively optimize compositions of HECs toward the maximized thermoelectric efficiency.^[^ ^43^ ^]^ Unlike conventional human‐guided selection, this model does not rely on expert heuristics but instead employs a stochastic approach to identify the most informative compositions for experimentation.^[^ ^44^, ^45^, ^46^ ^]^ The model iteratively assimilates composition‐property relationships, updating its predictions in response to new experimental data.^[^ ^47^ ^]^

Through six active learning iterations encompassing only 80 experiments, we successfully identified high‐performance compositions, demonstrating the effectiveness of an active learning‐driven optimization approach. As shown in Figure 1D, we discovered highly complex HECs with configurational entropy exceeding 18 J mol^−1^ K^−1^, a regime previously unexplored experimentally (See **Table** **2**). Moreover, these HECs exhibit significantly high *zT* values across broad temperature ranges, making them ideal candidates for improving thermoelectric conversion efficiency in power generation applications (Figure 1E).

**Table 2:** Peak *zT* values of previously reported high‐entropy thermoelectric alloys and the present work.

Refs.	Composition	Peak zT	T_peak_ [K]	Reported year
[53]	Pb_0.89_Sb_0.012_Sn_0.1_Se_0.5_Te_0.25_S_0.25_	1.8	900	2021
[18]	Ge_0.61_Ag_0.11_Sb_0.13_Pb_0.12_Bi_0.01_Te	2.7	750	2022
[54]	(Sn_0.74_Ge_0.2_Pb_0.1_)_0.75_Mn_0.275_Te	1.42	850	2018
[55]	Pb_0.935_Na_0.025_Cd_0.04_Se_0.5_S_0.25_Te_0.25_	2.0	900	2021
[56]	Ge_0.58_Sb_0.22_Te_0.8_(AgSnSe_2_)_0.2_	1.54	773	2021
[57]	(GeTe)_0.8_(AgSb_0.5_Bi_0.5_Te_2_)_0.2_	1.6	723	2023
[58]	Ga_0.025_(Sn_0.25_Pb_0.25_Mn_0.25_Ge_0.25_)_0.975_Te	1.52	823	2021
[59]	(GeSe)_0.5_(AgBiSe_2_)_0.5_	0.42	673	2018
[60]	Pb_0.94_SnTeSeLa_0.06_	0.8	873	2017
[61]	(Sn_0.5_Ge_0.4875_)_0.5_Pb_0.5_Te	1.61	773	2019
[62]	Ge_0.84_Pb_0.025_Sn_0.025_Sb_0.11_Te	2.3	723	2023
[63]	Ge_0.84_In_0.01_Pb_0.1_Sb_0.05_Te_0.997_I_0.003_	2.1	800	2019
[64]	Ge_0.4_Sn_0.4_Bi_0.02_Sb_0.12_Te	1.7	723	2020
[65]	GeAg_0.2_Sb_0.2_Se_1.4_	0.86	710	2017
[66]	AgSnSbSe_3_	1.14	723	2020
[67]	(GeTe)_0.8_(AgBiSe_2_)_0.2_	1.3	467	2019
[68]	(GeTe)_0.8_(AgSbSe_2_)_0.2_	1.9	660	2017
[69]	(Pb_0.15_Ge_0.85_Te)_0.8_(AgSbTe_2_)_0.2_	2.4	723	2022
[70]	AgMnSn_0.25_Pb_0.75_SbTe_4_	1.3	773	2022
[71]	(Sn_0.96_Sb_0.04_Te)_0.7_(Ge_0.5_Mn_0.5_Te)_0.3_	1.4	850	2022
[72]	Cd_0.02_(Sn_0.59_Pb_0.15_Ge_0.2_Sb_0.06_)_0.98_Te	1.5	800	2022
[73]	Ge_0.82_Sb_0.08_Te_0.9_(MnZnCdTe_3_)_0.1_	1.24	723	2022
This work	Pb_0.2_Ge_0.17_Sn_0.17_Cu_0.1_Ag_0.18_Sb_0.18_Se_0.5_Te_0.5_	2.35	473	2025

## 3 Entropically Stabilized Cubic‐Phase HECs

Our preliminary experiments revealed that even quinary alloys can undergo phase separation depending on the choice of alloying elements (Figure S1, Supporting Information). To mitigate this, we incorporated nine elements into a single material, increasing configurational entropy to promote a solid‐solution structure. This approach ensures a smooth and continuous variation of the objective function—the average quality factor (*B*_avg_), which will be discussed later—enhancing the performance and accuracy of the Gaussian process regression (GPR).

**Figure 2:**
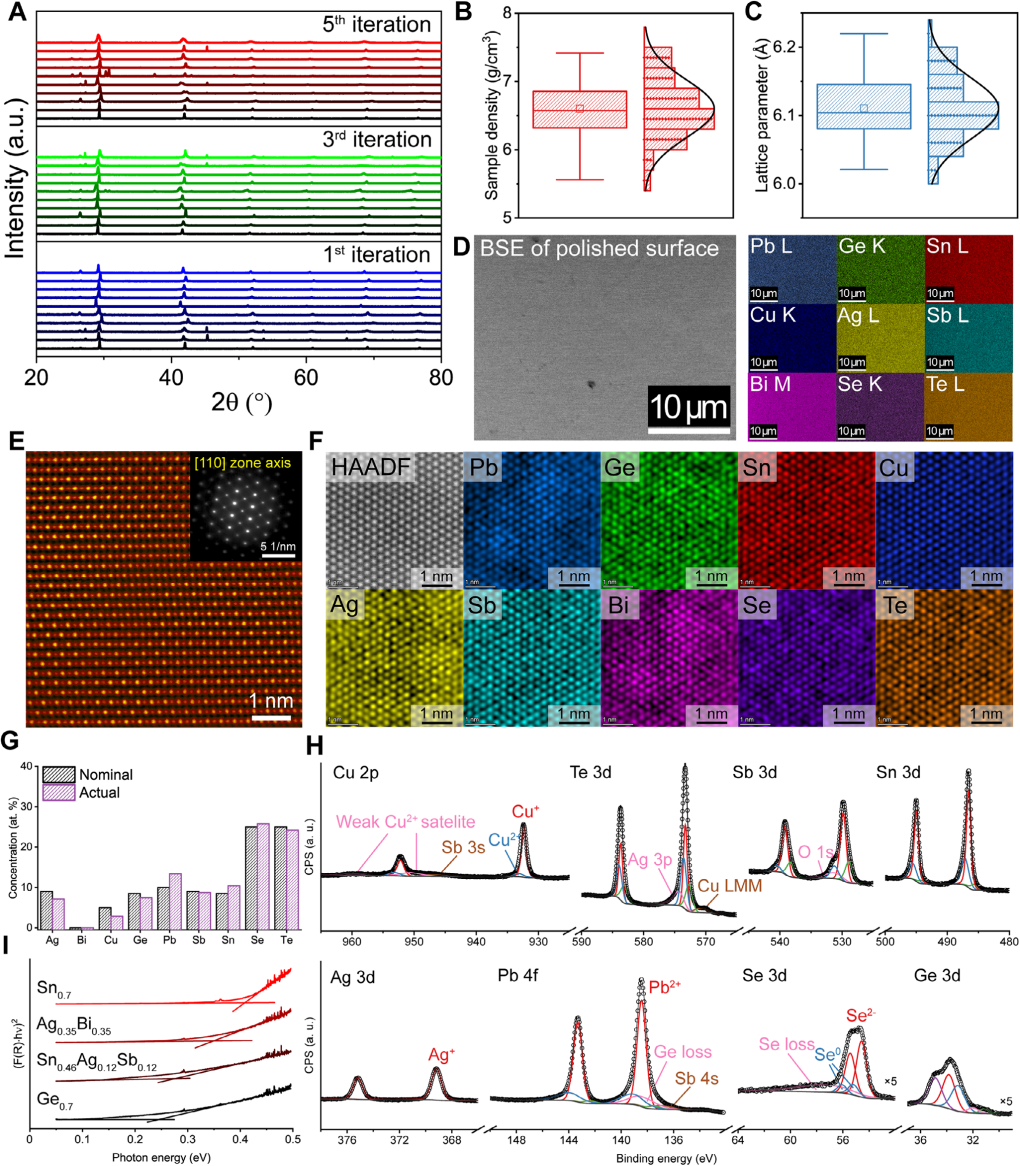
Emergence of high‐entropy phase. A) Powder XRD pattern of synthesized HECs for the 1st, 3rd, and 5th iteration, showing the stabilization of the single‐phase cubic structure. B,C) Distribution of sample density (B) and lattice parameter (C) with the compositional variation. Both exhibit Gaussian‐like variation, consistent with solid solution behavior. D) BSE‐SEM image with corresponding EDS elemental mapping of the nonary Pb_0.2_Ge_0.13_Sn_0.15_Cu_0.1_Ag_0.21_Bi_0.08_Sb_0.13_Se_0.5_Te_0.5_ alloy. All nine elements are uniformly distributed without segregation. E) Atomic‐resolution HAADF‐STEM images of the nonary alloy along the [110] zone axis. F) Atomic‐resolution EDS mapping of the nonary alloy along the [111] zone axis, showing homogeneous distribution of each element at the atomic scale. G,H) ICP‐OES elemental quantification (G) and XPS spectra (H) of the octonary Pb_0.2_Ge_0.17_Sn_0.17_Cu_0.1_Ag_0.18_Sb_0.18_Se_0.5_Te_0.5_ alloy. I) Tauc plot showing variation in the optical bandgap of HECs with composition.

**Figure** **2**A shows the powder X‐ray diffraction (PXRD) patterns of the synthesized HECs. Several initial training samples (blue curves) exhibited noticeable phase separation, often corresponding to endpoint compositions such as Pb_0.2_Ge_0.7_Cu_0.1_Se_0.5_Te_0.5_ (excluding Sn, Ag, Bi, and Sb), which were designed to explore material properties across a broad compositional space. However, phase stability improved significantly with the addition of more components, resulting in single‐phase cubic chalcogenides with the $Fm\bar{3}m$ space group. This enhanced stability is attributed to the entropic stabilization of high‐symmetry structures. Increasing the temperature (vibrational entropy) or the number of constituent components (configurational entropy) favors the cubic phase, as it provides more equivalent lattice sites for atomic species compared to low‐symmetry counterparts.^[^ ^74^, ^75^ ^]^ Consequently, polymorphic transformations upon heating–commonly observed in other metal chalcogenides such as GeTe, SnSe, and AgBiSe_2_–are effectively suppressed in HECs (Figures S2–S4, Supporting Information).

The single‐phase nature of HECs ensures a smooth and continuous variation in thermoelectric properties with composition, especially at elevated temperatures. Furthermore, being within the single‐phase regime guarantees a conventional solid‐solution‐like variation of properties with compositional changes, enhancing the predictability and design potential of HECs for thermoelectric applications. This behavior is evidenced by the Gaussian distribution of both sample density (Figure 2B) and the lattice parameters (Figure 2C) across the composition space. Scanning electron microscopy (SEM) analysis of the nonary Pb_0.2_Ge_0.13_Sn_0.15_Cu_0.1_Ag_0.21_Bi_0.08_Sb_0.13_Se_0.5_Te_0.5_ alloy, using backscattered electron (BSE) imaging and energy‐dispersive X‐ray spectroscopy (EDS), confirmed a homogeneous distribution of constituent elements within the matrix (Figure 2D).

Further structural analysis using scanning transmission electron microscopy (STEM) revealed additional insights into HECs’ crystallographic characteristics. High‐angle annular dark‐field (HAADF)‐STEM imaging and selected area electron diffraction (SAED) pattern along the [110] zone axis (Figure 2E) confirmed a rock salt structure. Notably, significant local variations in HAADF contrast were observed, likely indicating mixed atomic occupation in the cation/anion sublattices.^[^ ^76^ ^]^ Atomic‐resolution EDS mapping along the [111] zone axis (Figure 2F) further validated i) the homogeneous distribution of the constituent elements and ii) local fluctuation in atomic occupation.

Building on this structural analysis, we further investigated the elemental composition and bonding characteristics of HECs. Inductively coupled plasma‐optical emission spectroscopy (ICP‐OES) (Figure 2G) revealed slight deviations (within 3 at. %p) from nominal compositions, likely due to experimental artifacts during the synthesis process. However, most compositions remained within the expected range. Then, the bonding characteristics of the constituent elements of the HECs were studied using X‐ray photoelectron spectroscopy (XPS). The deconvoluted XPS spectra (Figure 2H) confirmed simultaneous detection of multiple elements, with complex valence states reflecting dynamic bonding configurations and random atomic occupation. These bonding characteristics play a critical role in enabling novel transport properties that are not achievable in conventional thermoelectric materials, where properties are primarily governed by the bonding characteristics of the constituent elements. Moreover, HECs were identified as narrow‐gap semiconductors, with bandgaps tunable via compositional adjustments (Figure 2I). This tunability is particularly important, as the optimal operating temperature of a thermoelectric material is closely tied to its bandgap. Together, these characteristics make HEC an ideal model system for exploring novel thermoelectric materials using the GPR‐based active learning framework.

## 4 Evolution of the Active Learning Model with a Thermoelectric Descriptor

The dimensionless thermoelectric figure‐of‐merit (*zT*), defined as:

(1) $$zT=\frac{S^{2}\sigma T}{\kappa }$$

where *S* is the Seebeck coefficient, σ is the electrical conductivity, *T* is the absolute temperature, and κ is the thermal conductivity of a material, has long been considered a direct performance indicator of a thermoelectric material.^[^ ^1^, ^36^ ^]^ One major advantage of utilizing *zT* as a thermoelectric descriptor is its intuitive representation of thermoelectric efficiency. However, *zT* is highly sensitive to the Fermi level, requiring optimal doping to achieve maximum performance in a given material.^[^ ^12^ ^]^

To address this issue, we hypothesized that the descriptor for the GPR‐based active learning should be highly robust to variations in the Fermi level (i.e., carrier concentration) to ensure reliable and efficient discovery of unexplored thermoelectric materials. This hypothesis is supported by previous studies showing that thermoelectric properties can vary significantly due to slight deviations in sample stoichiometry, synthesis parameters, and precursor purity, often resulting in carrier concentration changes on the order of magnitude.^[^ ^31^, ^77^ ^]^ Consequently, if the active learning model were trained using *zT* as a descriptor, its exploration decisions might be biased toward optimally doped samples while potentially overlooking promising candidates.

**Figure 3:**
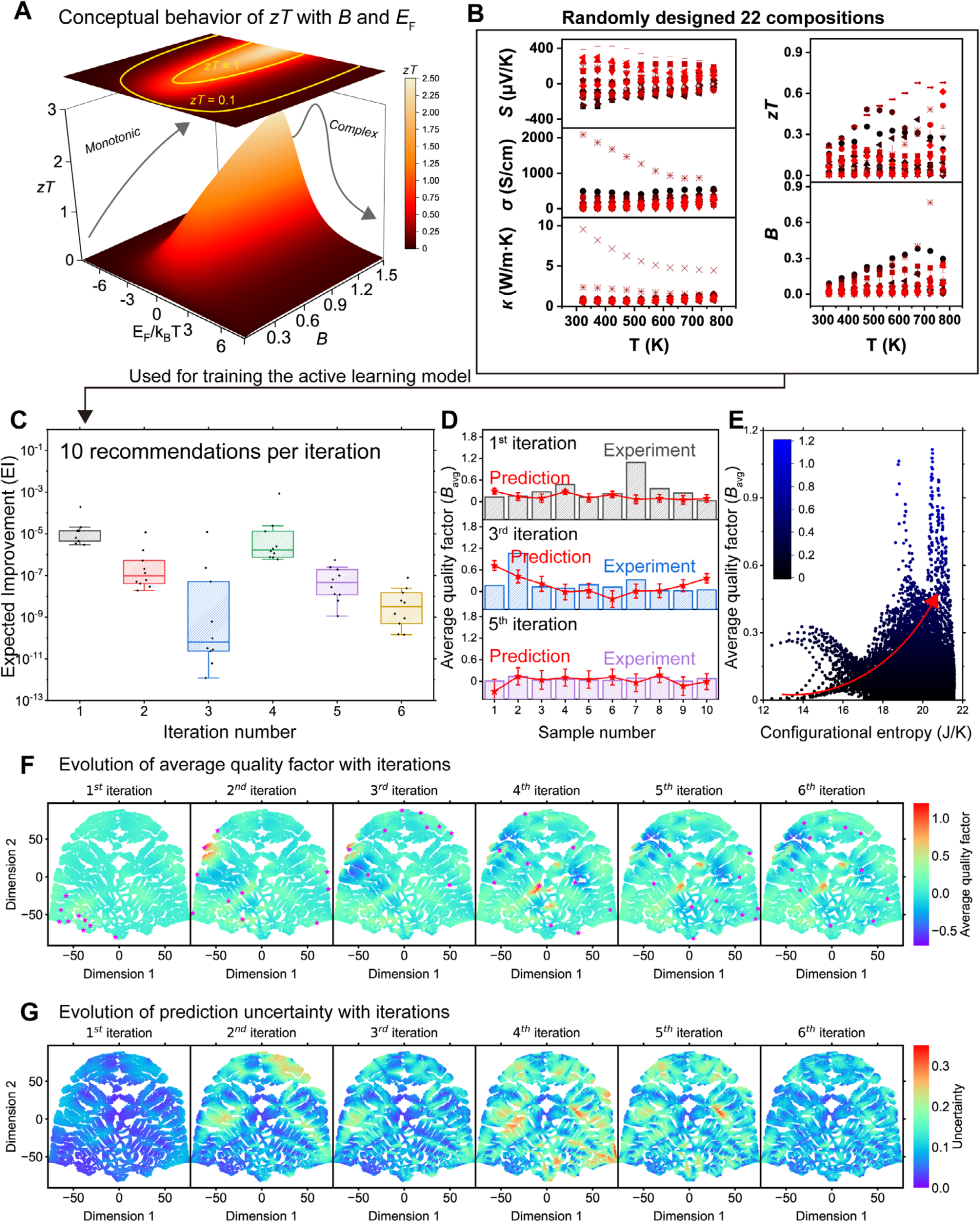
Active learning with physics‐informed descriptors. A) Conceptual illustration of the relationship between *zT*, reduced Fermi level (*E*_F_/*k*_B_ *T*), and thermoelectric quality factor (*B*) modeled under the single parabolic band approximation with deformation potential scattering. While *zT* exhibits a highly complex dependence on the Fermi level (i.e., carrier concentration), the monotonic relationship between *zT* and *B* simplifies the optimization of thermoelectric properties in the active learning process. B) Thermoelectric properties of 22 randomly designed samples with different compositions, showing broad variability of transport properties across different compositions. The average quality factor (*B*_avg_) of these samples was incorporated into the active learning model as an initial dataset. C) Variation of the expected improvement (EI) values during the iterative active learning sequence. D) Predicted and experimental *B*_avg_ values of 10 samples in the 1st, 3rd, and 5th iterations. E) Correlation between model‐predicted *B*_avg_ and configurational entropy. Higher entropy compositions generally yield higher *B*_avg_. F,G) t‐SNE representation of the design space, showing *B*_avg_ (F) and uncertainty (G) values at each iteration. The magenta symbols in (F) indicate compositions selected for experimental observations based on the query strategy. These maps visualize how the design space is progressively explored, with high *B*_avg_ regions and uncertain areas clearly distinguished.

To overcome this limitation, we employed the thermoelectric quality factor (*B*, see Supporting Information for calculation details) as a key descriptor for identifying materials with potentially high *zT*, assuming it can be optimally doped (**Figure** **3**A). *B* is known to be a robust indicator of both electronic and thermal properties, ensuring that improvements in *B* monotonically increase the maximum achievable *zT*.^[^ ^78^ ^]^ Consequently, even materials that appear underperforming in *zT* due to suboptimal doping can contribute valuable training data for the active learning model.

We initiated the active learning process with an initial dataset of 22 randomly designed compositions (Figure 3B). Each sample underwent temperature‐dependent measurements of *S*, σ, and κ, from which we derived the *zT* and *B*. Notably, both positive and negative values of *S* were observed, indicating that HECs can exhibit either p‐ or n‐type behavior. However, rather than distinguishing between majority carrier types, we focused on optimizing overall thermoelectric performance. Since *B*, like other thermoelectric properties, varies with temperature, we calculated its average value (*B*_avg_) across the measurement temperature range for model training.

Over six active learning iterations, we synthesized and experimentally evaluated 80 samples. After each training cycle, we assessed the entire design space to estimate the expected improvement (EI), which is a Bayesian acquisition function that quantifies the potential for improvement over the current best observation, balancing exploitation and exploration.^[^ ^79^ ^]^ The 10 compositions with the highest EI were selected for the subsequent batch. EI decreased steadily as the active learning progressed, indicating improved model predictions for *B*_avg_. (Figure 3C). This trend was further confirmed in Figure 3D, which illustrates the diminishing discrepancy between predicted and experimentally measured *B*_avg_ values over successive iterations.

During the fourth iteration, a high‐performance composition lying outside the model's initial predictive framework was discovered, temporarily increasing EI and uncertainty. However, these fluctuations stabilized in subsequent iterations. By the sixth iteration, the model had successfully identified multiple compositions demonstrating superior performance across the entire temperature range, with no further significant variations in key indicators. Given this convergence, we concluded that additional training was unnecessary and terminated the active learning process.

Our model predictions identified two distinct regions of high‐performing compositions. The first region is entirely Bi‐free and either completely excludes Ge or contains it in only trace amounts (mole fraction < 0.05). This region, highlighted in red in the upper‐left corner of the second iteration (Figure 3F), suggests that minimizing Ge content benefits thermoelectric properties in the HEC system. The second region is also free of Bi but incorporates Ge, Sn, Ag, and Sb in approximately equimolar proportions, forming a composition cluster in the central area of the fourth iteration. Both regions are predicted to achieve *B*_avg_ values exceeding 1.0 across the target temperature range, comparable to state‐of‐the‐art thermoelectric materials such as GeTe,^[^ ^80^ ^]^ Cu_2_Se,^[^ ^81^ ^]^ and SnSe,^[^ ^82^ ^]^ underscoring their potential for high thermoelectric performance.

Building on the active learning model's predictions, we explored the relationship between *B*_avg_ and configurational entropy across the entire design space (Figure 3E). The analysis reveals a clear trend: *B*_avg_ increases with higher configurational entropy. This correlation provides fundamental insight into the design of high‐entropy compounds for thermoelectric applications, reinforcing their potential as high‐performance thermoelectric materials. Notably, this finding aligns with the second predicted region, where near‐equimolar compositions consistently yield elevated *B*_avg_ ​values, further validating the model's accuracy.

We also examined uncertainty across the design space at each iteration (Figure 3G). During early active learning iterations, uncertainty occasionally increased. Although our initial set of 22 samples was deliberately distributed across the design space—maximizing coverage by including widely spaced compositions and several endpoint compositions (with zero mole fraction for certain elements)—many regions remained underexplored. Consequently, predictions in these areas were less reliable. When the model ventured into these regions, it encountered previously unobserved *B*_avg_ values extending beyond the initial prediction range, leading to temporary spikes in uncertainty, particularly in the second and fourth iterations.

In later stages, particularly during the fifth and sixth iterations, the active learning model effectively reduced uncertainty. By strategically selecting points from high‐uncertainty regions, obtaining experimental values, and incorporating these data into subsequent training cycles, the model refined its predictions iteratively. This process of targeted exploration and retraining ultimately stabilized the model, leading to a consistent decrease in overall uncertainty across the design space.

## 5 Assimilating Latent Design Rules in HECs

**Figure 4:**
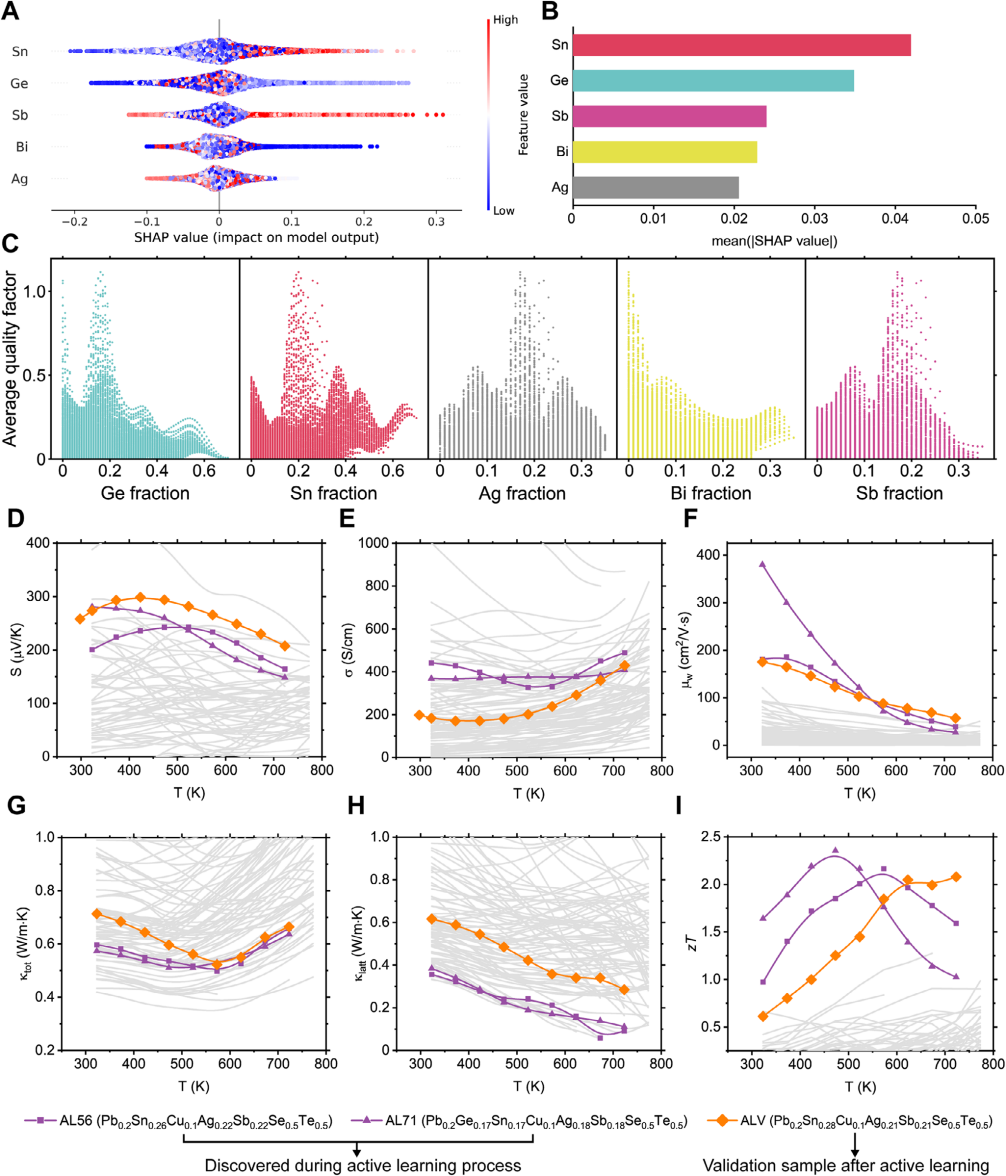
Latent thermoelectric design rule captured by the active learning. A) SHAP plot of the active learning model showing the impact of each element on *B*_avg_. B) Mean of the SHAP value plotted in (A). C) Contribution of five elements to *B*_avg_, indicating Bi is detrimental to the thermoelectric properties in this system, and an equal fraction of constituent elements is important. D–I) Experimentally measured Seebeck coefficient (D), electrical conductivity (E), weighted mobility (F), total thermal conductivity (G), lattice thermal conductivity (H), and *zT* values (I). Weighted mobility was calculated from the measured Seebeck coefficient and electrical conductivity. Gray lines denote experimental thermoelectric properties of HECs used to train the active learning model.

To quantitatively assess the contribution of each element in the complex nonary system, we employed SHapley Additive exPlanations (SHAP), a technique rooted in cooperative game theory that quantifies the contribution of each feature to the model's predictions.^[^ ^83^ ^]^ **Figure** **4**A visualizes the SHAP values for each element, ranking them vertically based on their cumulative influence across all samples. The horizontal axis represents individual SHAP values, indicating each element's impact on the model's prediction, while each point corresponds to a specific sample. The color scale (blue–red) reflects the mole fraction of the element, ranging from low to high. Notably, for Bi, blue points—corresponding to low Bi mole fractions—appear in the region of positive SHAP values, suggesting that reducing Bi content enhances the predicted *B_avg_*. Figure 4B further clarifies this trend by displaying the mean SHAP values, illustrating the overall importance of each element. From this perspective, Sn and Ge exhibit the strongest influence on *B*_avg_, followed by Sb, Bi, and Ag.

These findings align with Figure 4C, which depicts the predicted *B*_avg_ as a function of each element's mole fraction. While the true relationship between individual elements and *B_avg_* is highly complex, our active learning model identifies a clear trend: *B_avg_* tends to reach its optimal value when the mole fractions of most elements (except for Bi) are close to 0.2, forming an equimolar composition. As previously noted, increasing Bi content consistently reduces the expected *B_avg_*.

The exclusion of Bi from high‐performance compositions suggests that the active learning model inherently favors p‐type materials over n‐type materials in the HEC system, as Bi typically shifts the system toward n‐type conduction.^[^ ^8^, ^31^ ^]^ This observation aligns with established thermoelectric trends: base alloys in this HEC family, such as PbTe, SnTe, and GeTe, often exhibit significantly higher *zT* when doped to p‐type rather than n‐type.^[^ ^84^ ^]^ Similarly, AgSbSe_2_ and AgSbTe_2_ (both p‐type compounds)^[^ ^85^, ^86^ ^]^ generally outperform their n‐type counterparts, AgBiSe_2_ and AgBiTe_2_, in thermoelectric efficiency. Although these guidelines have long been recognized by researchers, they were never explicitly provided to the active learning model. This finding underscores the model's ability to extract fundamental design rules directly from experimental data, demonstrating its potential as a powerful tool for guiding thermoelectric material development.

Another notable finding is that high thermoelectric performance can be achieved even when Ge is completely excluded. At first glance, this result may seem counterintuitive, given that GeTe and many Ge‐containing thermoelectric alloys are well known for their high *zT* values. A previous study demonstrated that adding Ge to PbTe beyond its solubility limit can further enhance thermoelectric properties.^[^ ^87^ ^]^ However, the identification of high‐performance compositions without any Ge by our active learning model suggests the presence of alternative mechanisms or synergies that compensate for its absence. This result is particularly intriguing in the context of the complex interplay between electron and phonon transport in disordered systems, highlighting the potential for unconventional design strategies in high‐entropy thermoelectric materials.

A plausible explanation for this observation is that the model identifies compositions with an “optimal degree of disorder,” leveraging an effect reminiscent of Anderson localization.^[^ ^85^, ^88^ ^]^ In the previous section, many of the best‐performing samples exhibit Δ*S*_conf_ clustering ≈20.5 J mol^−1^ K^−1^, while the overall range of Δ*S*_conf_ in the design space spans from 13.5 to 21.7 J mol^−1^ K^−1^. While higher Δ*S*_conf_ is generally assumed to enhance thermoelectric properties,^[^ ^89^ ^]^ our active learning model—without pre‐training specific to high‐entropy alloys—avoided excessive incorporation of certain elements. This choice implies that the model autonomously inferred how excessive disorder could be detrimental by extracting latent insights from the experimental data.

Building on the active learning model's predictions, we analyzed the experimental thermoelectric properties of the HECs. To compare model‐guided discoveries with post‐training explorations, we plotted high‐performance compositions identified during active learning (in purple) alongside a composition synthesized after model training was completed (in orange). As shown in Figure 4D, the Seebeck coefficients of these high‐performance compositions range from 200 to 300 µV K^−1^, confirming p‐type semiconductor behavior with high carrier concentrations. Their electrical conductivities were moderate, spanning 200–400 S cm^−1^ at 323 K and increasing to ≈400–500 S cm^−1^ at 723 K (Figure 4E). Interestingly, these samples deviate from the common electronic transport behavior described by the single parabolic band model of degenerate semiconductors.^[^ ^90^ ^]^ The electronic and thermal properties of these disordered (or complex) alloys exhibit an unusual temperature dependence, which extends beyond the scope of this study and warrants further investigation.^[^ ^91^ ^]^

Our analysis revealed that the temperature‐dependent weighted mobility resembles the behavior expected when acoustic phonon scattering dominates electronic transport (Figure 4F).^[^ ^92^ ^]^ Additionally, the high‐performance samples exhibit glass‐like thermal conductivities, with total thermal conductivity ranging from 0.5 to 0.7 W m^−1^ K^−1^ across the measured temperature range (Figure 4G). We estimated the lattice contribution to thermal conductivity (Figure 4H) by subtracting the electronic thermal conductivity from the total thermal conductivity. While it is known that the Lorenz number extracted from the measured Seebeck coefficient can lead to anomalously high electronic thermal conductivity,^[^ ^93^, ^94^ ^]^ we confirmed that it follows the expected behavior of acoustic phonon scattering. Further estimation of the isothermal Lorenz number may be necessary for a more accurate determination of the lattice thermal conductivity of the HECs.

Ultimately, the experimentally measured *zT* values of the high‐performance compositions significantly surpass those of other compositions used during model training. Specifically, the AL56, AL71, and ALV samples achieved maximum *zT* values of 2.1, 2.3, and 2.1 at 573, 473, and 723 K, respectively. While these peak *zT* values are remarkable, an equally significant advantage of the HEC system is its ability to optimize the temperature at which maximum *zT* appears. This tunability, enabled through precise compositional engineering of the constituent elements, offers a strategic advantage for designing segmented thermoelectric legs to enhance overall thermoelectric conversion efficiency.

Taken together, these examples illustrate the active learning model's ability to discover novel, high‐performing compositions that mirror established design heuristics while also revealing new insights. Notably, with just 80 experimental samples across six active learning iterations, the model identified several compositions predicted to have high *B_avg_*. Subsequent measurements confirmed that three of these indeed exceed *zT* = 2 from 323 to 723 K. Achieving such results by brute force, given more than 16 000 possibilities in the design space, would be prohibitively time‐consuming and resource‐intensive. In addition, the active learning process not only pinpointed these exceptional thermoelectric materials but also provided a robust overview of the system's behavior, as reflected by *B_avg_* predictions covering the entire design space. By directing experimental efforts to the most promising regions, this active learning strategy greatly reduces the trial‐and‐error burden compared to conventional approaches, underscoring its efficiency for materials discovery.

## 6 Characterization of High‐Performance HECs

**Figure 5:**
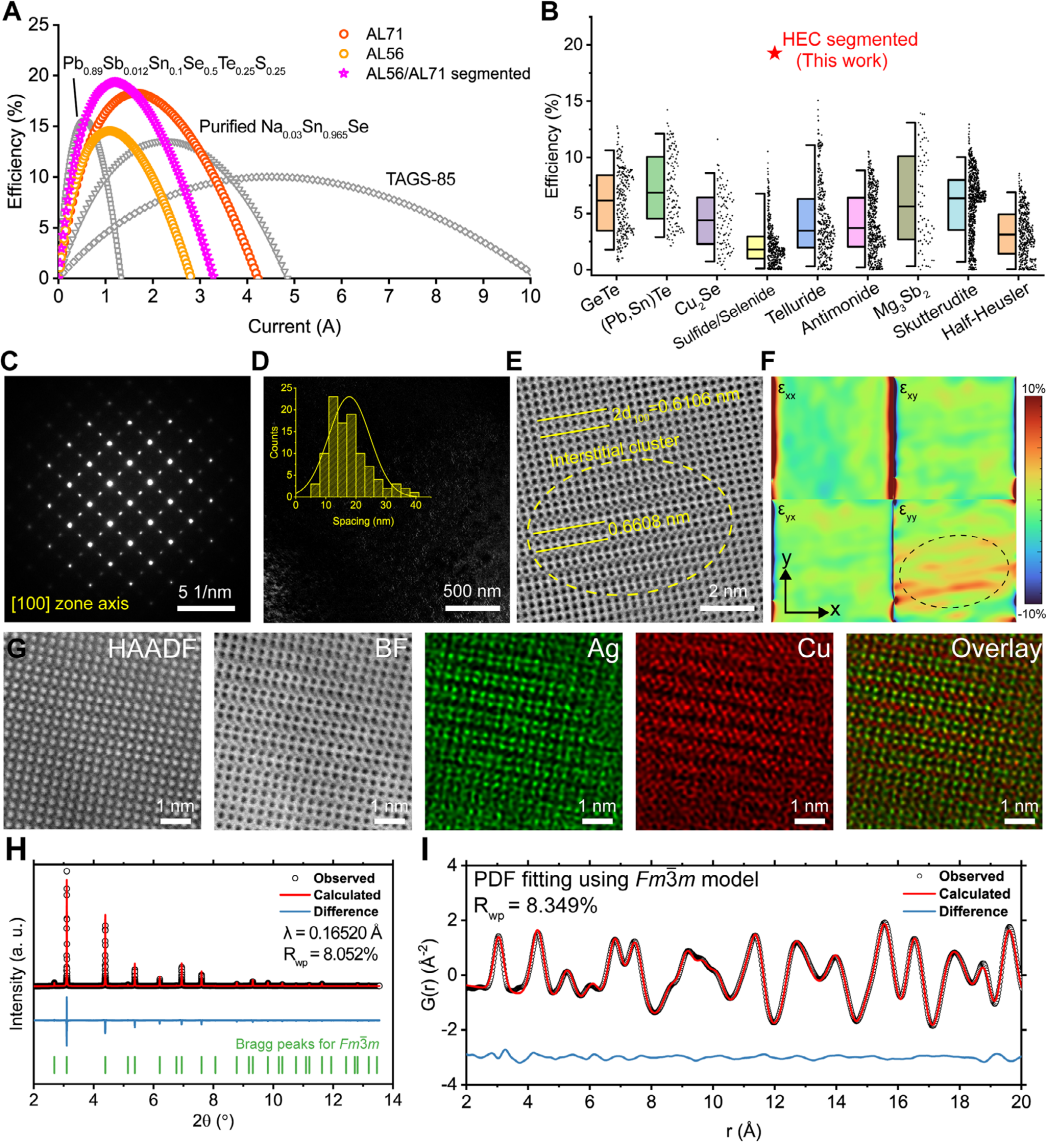
Characterization of discovered high‐performance HEC. A) Calculated thermoelectric conversion efficiency of representative thermoelectric materials and HECs. B) Comparison of the thermoelectric efficiency of various thermoelectric materials and HEC. C) Selected area electron diffraction pattern acquired along the [100] zone axis, showing unit cell doubling. D) DF‐TEM image obtained using the $\frac{1}{2}$(001) reflection. Inset is the distribution of the spacing between bright spots. E) Atomic‐resolution BF‐STEM image showing interstitial cluster region with lattice expansion. F) GPA strain mapping results showing significant tensile lattice strain along the y‐direction. G) Atomic‐resolution HAADF‐, BF‐STEM, and EDS mapping on Ag and Cu. The interstitial cluster mainly consists of Cu atoms. H,I) Synchrotron powder XRD (H) and total scattering‐pair distribution function (I) of the AL56 sample.

Experimental measurements confirmed that the discovered HECs exhibit exceptionally high *zT* values across a broad temperature range rather than at a single temperature point. This is particularly advantageous for thermoelectric applications, as thermoelectric elements experience a temperature gradient along their height, necessitating optimal *zT* across a wide operating range. **Figure** **5**A presents the theoretical conversion efficiencies of various state‐of‐the‐art thermoelectric materials, including TAGS‐85,^[^ ^95^ ^]^ purified SnSe,^[^ ^96^ ^]^ and lead chalcogenides,^[^ ^16^ ^]^ assuming a hot‐side temperature of 723 K and a cold‐side temperature of 323 K. Among them, purified Na_0.03_Sn_0.965_Se, a record‐holding material with *zT* = 3.1 at 783 K, achieves a peak efficiency of ≈13.55% under optimized conditions.^[^ ^96^ ^]^ However, AL56 is predicted to achieve 14.54% efficiency, surpassing purified Na_0.03_Sn_0.965_Se. Moreover, AL71 exhibits an even higher efficiency of 18.25%, underscoring the importance of low‐temperature *zT* in improving thermoelectric module performance.

Fabricating a segmented thermoelectric element using AL56 and AL71 could further enhance efficiency to 19.32%, assuming an ideal interface between the two materials. For comparison, Figure 5B presents a comprehensive dataset of theoretical conversion efficiencies for various thermoelectric materials under identical calculation conditions. These values, computed by Ryu et al.,^[^ ^97^ ^]^ are based on experimentally measured thermoelectric properties reported in the Starrydata2 thermoelectric database (as of November 24, 2021).^[^ ^98^ ^]^ High‐performance thermoelectric materials, such as GeTe, (Pb,Sn)Te, Mg_3_Sb_2_, tellurides, and skutterudites, have previously achieved efficiency records of ≈15%, outperforming Cu_2_Se, sulfide/selenide, antimonide, and half‐Heusler compounds. However, segmented HECs surpass these conventional materials due to their unprecedented compositional tunability and design flexibility, enabled by active learning‐guided discovery.

To investigate the mechanisms underlying this exceptional performance, we conducted atomic‐scale characterizations of the high‐performance samples. Figure 5C shows the SAED pattern of the AL56 sample observed along the [100] zone axis, revealing additional diffraction spots not expected in the $Fm\bar{3}m$ structure. Dark‐field TEM imaging using these extra spots (Figure 5D) revealed highly dense nanoscale streaks, evenly distributed throughout the sample with separations of 10–40 nm. This region consists of a superlattice structure formed by interstitial atoms embedded within the $Fm\bar{3}m$ lattice, where interstitial atoms appear periodically within the atomic columns in an alternating manner (Figure 5E). The presence of interstitial clusters induces local tensile strain, increasing the (100) interplanar distance from 6.106 to 6.608 Å, as confirmed by geometric phase analysis (GPA) strain mapping (Figure 5F). Atomic‐resolution EDS mapping (Figure 5G) reveals that Ag atoms primarily occupy the cation sublattice, while Cu atoms preferentially form interstitial clusters. These unconventional defect structures are expected to significantly reduce lattice thermal conductivity, contributing to the observed high *zT* values.

To further validate the structural characteristics, we conducted synchrotron PXRD (SPXRD) and total scattering‐pair distribution function (TS‐PDF) analyses. The high‐resolution SPXRD pattern (Figure 5H) closely matches the predicted $Fm\bar{3}m$ Bragg peak positions, confirming the formation of a highly symmetric phase. Furthermore, TS‐PDF analysis confirms that the local environment in HECs is well described by the $Fm\bar{3}m$ structure model (Figure 5I). In conventional cubic rock salt chalcogenides, local off‐centering of cations often results in significantly asymmetric first‐nearest‐neighbor peaks in the TS‐PDF pattern.^[^ ^99^, ^100^ ^]^ However, such asymmetry is absent in the HEC system near *r* = 3.0 Å, suggesting that high configurational entropy effectively stabilizes the symmetric $Fm\bar{3}m$ structure. This reduced structural distortion delocalizes the distribution of electrons, facilitating electron transport to improve charge carrier mobility.^[^ ^18^ ^]^ Additionally, the high‐symmetry configuration promotes band convergence, which is crucial for maintaining a high Seebeck coefficient alongside high electrical conductivity.^[^ ^101^ ^]^

## 7 Conclusion

While machine learning has enabled advances in developing functional high‐entropy materials, its application to thermoelectrics has remained challenging due to high experimental costs, limited training data, and the absence of suitable descriptors. In addition, the strong temperature dependence of thermoelectric properties further complicates the selection of a single descriptor that captures all relevant features. In this work, we addressed these challenges by stabilizing the high‐symmetry cubic phase, selecting Bavg as a physics‐informed descriptor, and implementing GPR for the surrogate model. These choices simplified the optimization process, allowing us to identify three high‐performance HECs with *zT* >2 while performing only 80 experiments out of 16206 possible combinations through a Bayesian active learning framework. This process was completed in just a few months, with a single experimentalist conducting melting, sintering, and measurements. Notably, this approach not only reduced the required manpower but also minimized potential experimental artifacts associated with multiple researchers. Beyond optimizing thermoelectric performance, our findings highlight the potential for discovering unprecedented HECs with novel constituent elements. The observed structural features and transport properties suggest the existence of previously unidentified physical phenomena unique to high‐entropy systems. These insights could provide a deeper understanding of electron and phonon transport in disordered systems, further advancing the design of next‐generation thermoelectric materials.

## Conflict of Interest

The authors declare no conflict of interest.

## Supporting information



Supporting Information

## Data Availability

The data that support the findings of this study are available from the corresponding author upon reasonable request.

## References

[1] G. J. Snyder , E. S. Toberer , Nat. Mater. 2008, 7, 105. 18219332 10.1038/nmat2090

[2] L. E. Bell , Science 2008, 321, 1457. 18787160 10.1126/science.1158899

[3] J. Yoon , H. Jang , M.‐W. Oh , T. Hilberath , F. Hollmann , Y. S. Jung , C. B. Park , Nat. Commun. 2022, 13, 3741. 35768427 10.1038/s41467-022-31363-8 PMC9243031

[4] Y. Liu , H.‐Y. Cheng , J. A. Malen , F. Xiong , Nat. Commun. 2024, 15, 4275. 38769104 10.1038/s41467-024-48583-9 PMC11106063

[5] T. Zhu , Y. Liu , C. Fu , J. P. Heremans , J. G. Snyder , X. Zhao , Adv. Mater. 2017, 29, 1605884.

[6] K. Biswas , J. He , I. D. Blum , C.‐I. Wu , T. P. Hogan , D. N. Seidman , V. P. Dravid , M. G. Kanatzidis , Nature 2012, 489, 414. 22996556 10.1038/nature11439

[7] L. i.‐D. Zhao , J. He , D. Berardan , Y. Lin , J.‐F. Li , C. e.‐W. Nan , N. Dragoe , Energy Environ. Sci. 2014, 7, 2900.

[8] J. Mao , H. Zhu , Z. Ding , Z. Liu , G. A. Gamage , G. Chen , Z. Ren , Science 2019, 365, 495. 31320557 10.1126/science.aax7792

[9] C. Fu , S. Bai , Y. Liu , Y. Tang , L. Chen , X. Zhao , T. Zhu , Nat. Commun. 2015, 6, 8144. 26330371 10.1038/ncomms9144 PMC4569725

[10] Y. Tang , Z. M. Gibbs , L. A. Agapito , G. Li , H.‐S. Kim , M. B. Nardelli , S. Curtarolo , G. J. Snyder , Nat. Mater. 2015, 14, 1223. 26436339 10.1038/nmat4430

[11] Y. Tang , R. Hanus , S. W. Chen , G. J. Snyder , Nat. Commun. 2015, 6, 7584. 26189943 10.1038/ncomms8584 PMC4518255

[12] Y. Pei , A. D. LaLonde , N. A. Heinz , X. Shi , S. Iwanaga , H. Wang , L. Chen , G. J. Snyder , Adv. Mater. 2011, 23, 5674. 22052689 10.1002/adma.201103153

[13] E. P. George , D. Raabe , R. O. Ritchie , Nat. Rev. Mater. 2019, 4, 515.

[14] S. Liu , D. i. Wu , M. Kong , W. u. Wang , L. Xie , J. He , ACS Energy Lett. 2025, 10, 925.

[15] Q. Tang , B. Jiang , K. Wang , W. Wang , B. Jia , T. Ding , Z. Huang , Y. Lin , J. He , Joule 2024, 8, 1641.

[16] B. Jiang , Y. Yu , J. Cui , X. Liu , L. Xie , J. Liao , Q. Zhang , Y. i. Huang , S. Ning , B. Jia , B. Zhu , S. Bai , L. Chen , S. J. Pennycook , J. He , Science 2021, 3718, 830. 10.1126/science.abe1292 33602853

[17] S. Ghosh , A. Nozariasbmarz , H. Lee , L. Raman , S. Sharma , R. B. Smriti , D. Mandal , Y. Zhang , S. K. Karan , N. Liu , J. L. Gray , M. Sanghadasa , S. Priya , W. Li , B. Poudel , Joule 2024, 8, 3303.

[18] B. Jiang , W. u. Wang , S. Liu , Y. Wang , C. Wang , Y. Chen , L. Xie , M. Huang , J. He , Science 2022, 377, 208. 35857539 10.1126/science.abq5815

[19] P. Koželj , S. Vrtnik , A. Jelen , S. Jazbec , Z. Jagličić , S. Maiti , M. Feuerbacher , W. Steurer , J. Dolinšek , Phys. Rev. Lett. 2014, 113, 107001. 25238377 10.1103/PhysRevLett.113.107001

[20] M. R. Kasem , K. Hoshi , R. Jha , M. Katsuno , A. Yamashita , Y. Goto , T. D. Matsuda , Y. Aoki , Y. Mizuguchi , Appl. Phys. Exp. 2020, 13, 033001.

[21] A. Yamashita , R. Jha , Y. Goto , T. D. Matsuda , Y. Aokia , Y. Mizuguchia , Dalton Trans. 2020, 49, 9118. 32573634 10.1039/d0dt01880e

[22] Z. M. Li , F. Körmann , B. Grabowski , J. Neugebauer , D. Raabe , Acta Mater. 2017, 136, 262.

[23] L. Han , F. Maccari , I. R. Souza Filho , N. J. Peter , Y. e. Wei , B. Gault , O. Gutfleisch , Z. Li , D. Raabe , Nature 2022, 608, 310. 35948715 10.1038/s41586-022-04935-3 PMC9365696

[24] Y. Yao , Z. Huang , P. Xie , S. D. Lacey , R. J. Jacob , H. Xie , F. Chen , A. Nie , T. Pu , M. Rehwoldt , D. Yu , M. R. Zachariah , C. Wang , R. Shahbazian‐Yassar , J. u. Li , L. Hu , Science 2018, 359, 1489. 29599236 10.1126/science.aan5412

[25] T. A. A. Batchelor , J. K. Pedersen , S. H. Winther , I. E. Castelli , K. W. Jacobsen , J. Rossmeisl , Joule 2019, 3, 834.

[26] M. Kim , M. Y. Ha , W.‐B. Jung , J. Yoon , E. Shin , I. l.‐D. Kim , W. B. o. Lee , Y. Kim , H.‐T. Jung , Adv. Mater. 2022, 34, 2108900. 10.1002/adma.202108900 35229377

[27] Y. e. Sheng , Y. Wu , J. Yang , W. Lu , P. Villars , W. Zhang , npj Comput. Mater. 2020, 6, 171.

[28] T. Zhu , R. He , S. Gong , T. Xie , P. Gorai , K. Nielsch , J. C. Grossman , Energy Environ. Sci. 2021, 14, 3559.

[29] G. Kim , H. Diao , C. Lee , A. T. Samaei , T. u. Phan , M. de Jong , K. e. An , D. Ma , P. K. Liaw , W. Chen , Acta Mater. 2019, 181, 124.

[30] Y. Gan , G. J. Wang , J. Zhou , Z. M. Sun , npj Comput. Mater. 2021, 7, 176.

[31] H. Jang , M. Y. Toriyama , S. Abbey , B. Frimpong , J. P. Male , G. J. Snyder , Y. S. Jung , M.‐W. Oh , Adv. Mater. 2022, 34, 2204132. 10.1002/adma.202204132 35944565

[32] W. Chen , J.‐H. Pöhls , G. Hautier , D. Broberg , S. Bajaj , U. Aydemir , Z. M. Gibbs , H. Zhu , M. Asta , G. J. Snyder , B. Meredig , M. A. White , K. Persson , A. Jain , J. Mater. Chem. C 2016, 4, 4414.

[33] A. J. Hong , Y. X. Tang , J. M. Liu , J. Phys. Chem. C 2021, 125, 24796.

[34] R. Li , X. Li , L. Xi , J. Yang , D. J. Singh , W. Zhang , ACS Appl. Mater. Interfaces 2019, 11, 24859. 31025850 10.1021/acsami.9b01196

[35] P. Gorai , V. Stevanovic , E. S. Toberer , Nat. Rev. Mater. 2017, 2, 17053.

[36] J. He , T. M. Tritt , Science 2017, 357, aak9997. 10.1126/science.aak9997 28963228

[37] H. Ming , Z. Z. Luo , Z. Zou , M. G. Kanatzidis , Chem. Rev. no. 2025, 125, 3932. 10.1021/acs.chemrev.4c00786 40105866

[38] S. Han , S. Dai , J. Ma , Q. Ren , C. Hu , Z. Gao , M. Duc Le , D. Sheptyakov , P. Miao , S. Torii , T. Kamiyama , C. Felser , J. Yang , C. Fu , T. Zhu , Nat. Phys. 2023, 19, 1649.

[39] L. Han , S. Zhu , Z. Rao , C. Scheu , D. Ponge , A. Ludwig , H. Zhang , O. Gutfleisch , H. Hahn , Z. Li , D. Raabe , Nat. Rev. Mater. 2024, 9, 846.

[40] T. J. Slade , K. Pal , J. A. Grovogui , T. P. Bailey , J. Male , J. F. Khoury , X. Zhou , D. Y. Chung , G. J. Snyder , C. Uher , V. P. Dravid , C. Wolverton , M. G. Kanatzidis , J. Am. Chem. Soc. 2020, 142, 12524. 32628474 10.1021/jacs.0c05650

[41] S. Zhou , J. Zhang , D. Liu , Z. Lin , Q. Huang , L. Bao , R. Ma , Y. Wei , Acta Mater. 2010, 58, 4978.

[42] J. K. i. Lee , B. Ryu , S. Park , J. i. H. Son , J. Park , J. Jang , M.‐W. Oh , S. Park , Acta Mater. 2022, 222, 117443.

[43] B. J. Shields , J. Stevens , J. Li , M. Parasram , F. Damani , J. I. M. Alvarado , J. M. Janey , R. P. Adams , A. G. Doyle , Nature 2021, 590, 89. 33536653 10.1038/s41586-021-03213-y

[44] Z. Rao , P. o.‐Y. Tung , R. Xie , Y. e. Wei , H. Zhang , A. Ferrari , T. P. C. Klaver , F. Körmann , P. T. Sukumar , A. Kwiatkowski da Silva , Y. Chen , Z. Li , D. Ponge , J. Neugebauer , O. Gutfleisch , S. Bauer , D. Raabe , Science 2022, 378, 78. 36201584 10.1126/science.abo4940

[45] M. Kim , Y. Kim , M. Y. Ha , E. Shin , S. J. Kwak , M. Park , I. l.‐D. Kim , W.‐B. Jung , W. B. o. Lee , Y. Kim , H.‐T. Jung , Adv. Mater. 2023, 35, 2211497. 10.1002/adma.202211497 36762586

[46] M. Park , M. Kim , Y. Kim , S. J. Kwak , W.‐B. Jung , H.‐T. Jung , W. B. Lee , Y. Kim , Chem. Eng. J. 2025, 518, 164693.

[47] J. Moon , W. Beker , M. Siek , J. Kim , H. S. Lee , T. Hyeon , B. A. Grzybowski , Nat. Mater. 2024, 23, 108. 37919351 10.1038/s41563-023-01707-w

[48] J. Carrete , W. Li , N. Mingo , S. D. Wang , S. Curtarolo , Phys. Rev. X 2014, 4, 011019.

[49] Q. i. Ren , D. Chen , L. Rao , Y. Lun , G. Tang , J. Hong , J. Mater. Chem. A 2024, 12, 1157.

[50] Z. L. Wang , Y. Yokoyama , T. Onda , Y. Adachi , Z. C. Chen , Adv. Electron. Mater. 2019, 5, 1900079.

[51] K. Song , G. Xu , A. N. M. Tanvir , K. e. Wang , M. d. O. Bappy , H. Yang , W. Shang , L. e. Zhou , A. W. Dowling , T. Luo , Y. Zhang , J. Mater. Chem. A 2024, 12, 21243.

[52] Y. Long , C. Zhong , X. Ma , J. Zhang , H. Yao , J. Liu , K. Hu , Q. Zhang , X. i. Lin , ACS Appl. Mater. Interfaces 2025, 17, 19856. 40110715 10.1021/acsami.4c19494

[53] B. Jiang , Y. Yu , J. Cui , X. Liu , L. Xie , J. Liao , Q. Zhang , Y. i. Huang , S. Ning , B. Jia , B. Zhu , S. Bai , L. Chen , S. J. Pennycook , J. He , Science 2021, 371, 830. 33602853 10.1126/science.abe1292

[54] L. Hu , Y. Zhang , H. Wu , J. Li , Y. u. Li , M. Mckenna , J. He , F. Liu , S. J. Pennycook , X. Zeng , Adv. Energy Mater. 2018, 8, 1802116.

[55] B. Jiang , Y. Yu , H. Chen , J. Cui , X. Liu , L. Xie , J. He , Nat. Commun. 2021, 12, 3234. 34050188 10.1038/s41467-021-23569-z PMC8163856

[56] G. Liang , T. Lyu , L. Hu , W. Qu , S. Zhi , J. Li , Y. Zhang , J. He , J. Li , F. Liu , C. Zhang , W. Ao , H. Xie , H. Wu , ACS Appl. Mater. Interfaces 2021, 13, 47081. 34565145 10.1021/acsami.1c14801

[57] J. Zhong , G. Liang , J. Cheng , W. Ao , C. Zhang , J. Li , F. Liu , S. Zhang , L. Hu , Sci. China Mater. 2023, 66, 696.

[58] X. Wang , H. Yao , Z. Zhang , X. Li , C. Chen , L. Yin , K. Hu , Y. Yan , Z. Li , B. Yu , F. Cao , X. Liu , X. Lin , Q. Zhang , ACS Appl. Mater. Interfaces 2021, 13, 18638. 33847476 10.1021/acsami.1c00221

[59] S. Roychowdhury , T. Ghosh , R. Arora , U. V. Waghmare , K. Biswas , Angew. Chem., Int. Ed. 2018, 57, 15167. 10.1002/anie.201809841 30225858

[60] Z. Fan , H. Wang , Y. Wu , X. Liu , Z. Lu , Mater. Res. Lett. 2017, 5, 187.

[61] S. Y. Zhao , R. Chen , J. Q. Li , L. Yang , C. H. Zhang , Y. Li , F. S. Liu , W. Q. Ao , J. Alloys Compd. 2019, 777, 1334.

[62] A. Das , P. Acharyya , S. Das , K. Biswas , J. Mater. Chem. A 2023, 11, 12793.

[63] Y. Qiu , Y. Jin , D. Wang , M. Guan , W. He , S. Peng , R. Liu , X. Gao , L. i.‐D. Zhao , J. Mater. Chem. A 2019, 7, 26393.

[64] A. Suwardi , J. Cao , L. Hu , F. Wei , J. Wu , Y. Zhao , S. u. H. Lim , L. Yang , X. Y. i. Tan , S. W. Chien , Y. Yin , W. u.‐X. Zhou , W. L. Mun Nancy , X. Wang , S. H. Lim , X. Ni , D. Li , Q. Yan , Y. Zheng , G. Zhang , J. Xu , J. Mater. Chem. A 2020, 8, 18880.

[65] Z. Huang , S. A. Miller , B. Ge , M. Yan , S. Anand , T. Wu , P. Nan , Y. Zhu , W. Zhuang , G. J. Snyder , P. Jiang , X. Bao , Angew. Chem., Int. Ed. 2017, 56, 14113. 10.1002/anie.201708134 28929555

[66] Y. Luo , S. Hao , S. Cai , T. J. Slade , Z. Z. Luo , V. P. Dravid , C. Wolverton , Q. Yan , M. G. Kanatzidis , J. Am. Chem. Soc. 2020, 142, 15187. 32786784 10.1021/jacs.0c07803

[67] M. Samanta , T. Ghosh , R. Arora , U. V. Waghmare , K. Biswas , J. Am. Chem. Soc. 2019, 141, 19505. 31735034 10.1021/jacs.9b11405

[68] M. Samanta , S. Roychowdhury , J. Ghatak , S. Perumal , K. Biswas , Chemistry – A European Journal 2017, 23, 7438. 28436062 10.1002/chem.201701480

[69] X. Wang , H. Yao , L. i. Yin , W. Xue , Z. Zhang , S. Duan , L. Chen , C. Chen , J. Sui , X. Liu , Y. Wang , J. Mao , Q. Zhang , X. i. Lin , Adv. Energy Mater. 2022, 12, 2201043.

[70] Z. Ma , Y. Luo , W. Li , T. Xu , Y. Wei , C. Li , A. Y. Haruna , Q. Jiang , D. Zhang , J. Yang , Chem. Mater. 2022, 34, 8959.

[71] Q. Zhang , R. Wang , K. Song , X. Tan , H. Hu , Z. Guo , G. Wu , P. Sun , G.‐Q. Liu , J. Jiang , Nano Energy 2022, 94, 106940.

[72] Q. Zhang , Z. Guo , R. Wang , X. Tan , K. Song , P. Sun , H. Hu , C. Cui , G.‐Q. Liu , J. Jiang , Adv. Funct. Mater. 2022, 32, 2205458.

[73] Y. Huang , S. Zhi , S. Zhang , W. Yao , W. Ao , C. Zhang , F. Liu , J. Li , L. Hu , Materials 2022, 15, 6798. 36234135 10.3390/ma15196798 PMC9572701

[74] S. S. Aamlid , M. Oudah , J. Rottler , A. M. Hallas , J. Am. Chem. Soc. 2023, 145, 5991. 36881986 10.1021/jacs.2c11608

[75] C. M. Rost , E. Sachet , T. Borman , A. Moballegh , E. C. Dickey , D. Hou , J. L. Jones , S. Curtarolo , J.‐P. Maria , Nat. Commun. 2015, 6, 8485. 26415623 10.1038/ncomms9485 PMC4598836

[76] W. u. Wang , S. Liu , Y. Wang , B. Jia , Y. i. Huang , L. Xie , B. Jiang , J. He , Sci. Adv. 2024, 10, adp4372. 10.1126/sciadv.adp4372 PMC11192076 38905337

[77] S. Huang , T.‐R. Wei , H. Chen , J. Xiao , M. Zhu , K. Zhao , X. Shi , ACS Appl. Mater. Interfaces 2021, 13, 60192. 34847670 10.1021/acsami.1c18483

[78] E. Witkoske , X. Wang , J. Maassen , M. Lundstrom , Mater. Today Phys. 2019, 8, 43.

[79] J. T. Wilson , F. Hutter , M. P. Deisenroth , in Proceedings of the 32nd International Conf. on Neural Information Processing Systems , Curran Associates Inc., Montréal, Canada 2018.

[80] A. Suwardi , J. Cao , Y. Zhao , J. Wu , S. W. Chien , X. Y. Tan , L. Hu , X. Wang , W. Wang , D. Li , Y. Yin , W.‐X. Zhou , D. V. M. Repaka , J. Chen , Y. Zheng , Q. Yan , G. Zhang , J. Xu , Mater. Today Phys. 2020, 14, 100239.

[81] K. Zhao , M. Guan , P. Qiu , A. B. Blichfeld , E. Eikeland , C. Zhu , D. Ren , F. Xu , B. o. B. Iversen , X. Shi , L. Chen , J. Mater. Chem. A 2018, 6, 6977.

[82] B. Qin , D. Wang , T. Hong , Y. Wang , D. Liu , Z. Wang , X. Gao , Z.‐H. Ge , L. i.‐D. Zhao , Nat. Commun. 2023, 14, 1366. 36914654 10.1038/s41467-023-37114-7 PMC10011372

[83] S. Mangalathu , S. H. Hwang , J. S. Jeon , Eng. Struct. 2020, 219, 110927.

[84] H. Zhu , J. Mao , Z. Feng , J. Sun , Q. Zhu , Z. Liu , D. J. Singh , Y. Wang , Z. Ren , Sci. Adv. 2019, 5, aav5813. 10.1126/sciadv.aav5813 PMC6588382 31245535

[85] S. Roychowdhury , T. Ghosh , R. Arora , M. Samanta , L. Xie , N. K. Singh , A. Soni , J. He , U. V. Waghmare , K. Biswas , Science 2021, 371, 722. 33574210 10.1126/science.abb3517

[86] S. N. Guin , A. Chatterjee , D. S. Negi , R. Datta , K. Biswas , Energy Environ. Sci. 2013, 6, 2603.

[87] P. Jood , M. Ohta , A. Yamamoto , M. G. Kanatzidis , Joule 2018, 2, 1339.

[88] M. T. Agne , F. R. L. Lange , J. P. Male , K. S. Siegert , H. Volker , C. Poltorak , A. Poitz , T. Siegrist , S. Maier , G. J. Snyder , M. Wuttig , Matter 2021, 4, 2970.

[89] R. Liu , H. Chen , K. Zhao , Y. Qin , B. Jiang , T. Zhang , G. Sha , X. Shi , C. Uher , W. Zhang , L. Chen , Adv. Mater. 2017, 29, 1702712. 10.1002/adma.201702712 28833741

[90] Y. Z. Pei , A. LaLonde , S. Iwanaga , G. J. Snyder , Energy Environ. Sci. 2011, 4, 2085.

[91] Y. Luo , S. Hao , S. Cai , T. J. Slade , Z. Z. Luo , V. P. Dravid , C. Wolverton , Q. Yan , M. G. Kanatzidis , J. Am. Chem. Soc. 2020, 142, 15187. 32786784 10.1021/jacs.0c07803

[92] G. J. Snyder , A. H. Snyder , M. Wood , R. Gurunathan , B. H. Snyder , C. Niu , Adv. Mater. 2020, 32, 2001537. 10.1002/adma.202001537 32410214

[93] O. Cherniushok , T. Parashchuk , G. J. Snyder , K. T. Wojciechowski , Adv. Mater. 2025, 37, 2420556. 40116527 10.1002/adma.202420556 PMC12051737

[94] H. S. Kim , Z. M. Gibbs , Y. L. Tang , H. Wang , G. J. Snyder , APL Mater. 2015, 3, 041506.

[95] E. M. Levin , S. L. Bud'ko , K. Schmidt‐Rohr , Adv. Funct. Mater. 2012, 22, 2766.

[96] C. Zhou , Y. K. Lee , Y. Yu , S. Byun , Z.‐Z. Luo , H. Lee , B. Ge , Y.‐L. Lee , X. Chen , J. i. Y. Lee , O. Cojocaru‐Mirédin , H. Chang , J. Im , S.‐P. Cho , M. Wuttig , V. P. Dravid , M. G. Kanatzidis , I. n. Chung , Nat. Mater. 2021, 20, 1378. 34341524 10.1038/s41563-021-01064-6 PMC8463294

[97] B. Ryu , J. Chung , M. Kumagai , T. Mato , Y. Ando , S. Gunji , A. Tanaka , D. Yana , M. Fujimoto , Y. Imai , Y. Katsura , S. Park , iScience 2023, 26, 106494. 37091247 10.1016/j.isci.2023.106494 PMC10114237

[98] Y. Katsura , M. Kumagai , T. Kodani , M. Kaneshige , Y. Ando , S. Gunji , Y. Imai , H. Ouchi , K. Tobita , K. Kimura , K. Tsuda , Sci. Technol. Adv. Mater. 2019, 20, 511.

[99] E. S. Božin , C. D. Malliakas , P. Souvatzis , T. Proffen , N. A. Spaldin , M. G. Kanatzidis , S. J. L. Billinge , Science 2010, 330, 1660. 21164012 10.1126/science.1192759

[100] M. Dutta , M. V. D. Prasad , J. Pandey , A. Soni , U. V. Waghmare , K. Biswas , Angew. Chem. Int, Ed. 2022, 61, 202200071. 10.1002/anie.202200071 35137508

[101] J. Zhang , R. Liu , N. Cheng , Y. Zhang , J. Yang , C. Uher , X. Shi , L. Chen , W. Zhang , Adv. Mater. 2014, 26, 3848. 24692165 10.1002/adma.201400058

