# Supplementary material for: Active Learning‐Guided Accelerated Discovery of Ultra‐Efficient High‐Entropy Thermoelectrics
Source: Adv Mater. 2025 Oct 9;38(10):e15054. doi: 10.1002/adma.202515054 (PMC12910547; doi:10.1002/adma.202515054)
Supplement: Supplementary file 1 — Supporting Information [file ADMA-38-e15054-s001.docx]

Supporting Information

**Active learning-guided accelerated discovery of ultra-efficient high-entropy thermoelectrics**

*Hanhwi Jang^1‡^, Wooseok Lee^2‡^, Hwa-Jung Kim^3^, Sohyang Cha^3^, Hosun Shin^3^, Won Bo Lee^2,4^, Min-Wook Oh^5^*, Yeon Sik Jung^1^*, YongJoo Kim^6^**

^1^Department of Materials Science and Engineering, Korea Advanced Institute of Science and Technology, Daejeon 34141, Republic of Korea

^2^School of Chemical and Biological Engineering, Seoul National University, Seoul 08826, Republic of Korea

^3^Division of Chemical and Materials Metrology, Korea Research Institute of Standards and Science (KRISS), Daejeon 34113, Republic of Korea

^4^School of Transdisciplinary Innovations, Seoul National University, Seoul 08826, Republic of Korea

^5^Department of Materials Science and Engineering, Hanbat National University, Daejeon 34158, Republic of Korea

^6^Department of Materials Science and Engineering, Korea University, Seoul 02841, Republic of Korea

‡These authors contributed equally to this work: Hanhwi Jang and Wooseok Lee

*Corresponding authors. Email: mwoh@hanbat.ac.kr, ysjung@kaist.ac.kr, cjyjee@korea.ac.kr

Materials and Methods

Synthesis

Thermoelectric high entropy alloys (HEAs) were synthesized by vacuum arc melting method (MAM-1, Edmund Buhler). Pb shots (99.99%, Alfa Aesar), Ge shots (99.99%, American Elements), Sn shots (99.999%, American Elements), Cu pellets (99.997%, Yoochang Metal), Ag shots (99.99%, Alfa Aesar), Bi shots (99.999%, 5N Plus), Sb shots (99.999%, 5N Plus), Se shots (99.999%, 5N Plus), Te shots (99.999%, 5N Plus) were used as received without any purification. The elements were weighed according to the stoichiometric ratio and placed in a water-cooled copper crucible. The chamber was evacuated with a built-in roughing pump and purged three times with high purity Ar gas (99.999%). Then, the electric arc was generated by applying a high voltage to a tungsten electrode under an Ar atmosphere. The melting process was repeated at least three times, and the resulting button was reversed each time to ensure the homogeneous melting of the sample. The mass loss of the final product is less than 5% of the initial mass; therefore, we did not add an excess amount of the starting material to compensate for the loss due to sample evaporation.

After the arc melting process, the sample was pulverized using agate mortar and pestle and the powder was sieved below 45 μm. Then, the powder was placed in a graphite mold with a diameter of 12.7 mm and pressed with a graphite punch having a same diameter. The densification process was performed by spark plasma sintering (SPS, Dr. Sinter SPS-211, Fuji Electronic Industrial) at 773 K for ten minutes under a uniaxial pressure of 50 MPa. The chamber was evacuated with a built-in roughing pump and purged three times with high purity Ar gas (99.999%) prior to sintering.

When designing the composition of nonary HEAs consisting of Pb, Ge, Sn, Cu, Ag, Sb, Bi, Se, and Te, we deliberately constrained the degrees of freedom to reduce the size of the design space, as follows:

1. The molar fractions of cations and anions were normalized such that $\sum x_{cations}=\sum x_{anions}=1$, where $x_{i}$ denotes the molar fraction of the *i*-th element.
2. The molar fractions of Se and Te were fixed at $x_{Se}=x_{Te}=0.5$ to maximize configurational entropy and suppress the formation of secondary phases.
3. $x_{Pb}$was fixed at 0.2 to promote cubic phase stability, as Pb-based chalcogenides (PbSe or PbTe) exhibit stable cubic structures without phase transitions, unlike other metal chalcogenides.
4. $x_{Cu}$ was fixed at 0.1 due to its limited solubility in binary metal chalcogenides with cubic structures.
5. The Ag concentration was constrained by $x_{Ag}=x_{Bi}+x_{Sb}$ to maintain the stoichiometry of I-V-VI_2_ ternary cubic chalcogenides.

The molar fraction of constituent elements were varied in incremenents of 1%, resulting in a design space of approximately 16,206 possible compositions.

Thermoelectric property measurement

The sintered sample was cut into a bar shape (3 mm × 3 mm × 8 mm^2^) and cylindrical shape (ϕ12.7 × 2 mm) for the measurement of electrical and thermal properties, respectively. The Seebeck coefficient and electrical conductivity were measured simultaneously using a commercial system (ZEM-3, ULVAC) equipped with R-type thermocouples. The chamber was evacuated and purged with high purity He (99.999%) gas. The measurement was then performed under a weak He backpressure to minimize the sample outgassing. The Seebeck coefficient was determined by measuring the thermopower under a temperature gradient, which was given by Joule heating of a gradient heater located at the bottom electrode. Although the temperature difference between the top and the bottom electrodes was 0, 10, 20, and 30 K, the actual temperature difference in a sample was deliberately controlled to not to exceed 5 K to eliminate the possible error in the Seebeck coefficient measurement from uneven temperature distribution in the sample. Thermal conductivity was estimated from the product of the density (ρ), specific heat capacity (C_p_), and thermal diffusivity (D). The thermal diffusivity was measured by the laser flash method (LFA 467, Netzsch. Ltd.). The specific heat capacity was estimated using the Dulong-Petit law. The samples were coated with a thin graphite layer to increase the emissivity. Density was measured by the immersion method in deionized water. The reliability of the measurement apparatus confirmed using the standard sample. A constantan alloy (Cu_55_Ni_45_) and Pyroceram 9606 (SiO_2_–Al_2_O_3_–MgO–TiO_2_ composite) were used to calibrate the Seebeck coefficient & electrical conductivity measurement equipment and thermal diffusivity measurement devices, respectively, which showed excellent agreement with the literature values with uncertainty less than 10%.

Characterization

The specimen for transmission electron microscopy (TEM) analysis was prepared by a focused ion beam (FIB, Helios G4, FEI Company, USA) with liquid gallium metal as an ion source. The specimen was milled to the final thickness of approximately 40 nm to ensure the electron transparency. The TEM imaging was conducted by spherical aberration-corrected TEM (Spectra Ultra, FEI Company) at 300 kV equipped with S-CORR spherical aberration corrector. To minimize electron drift, the measurement was started at 24 h after the sample loading. Energy-dispersive X-ray spectroscopy (EDX) elemental mapping was performed with Ultra-X silicon-drift EDX detectors under an acceleration voltage of 200 kV to reduce specimen damage and improve the EDS signal. The background noise in EDS signal was reduced by applying Wiener filter. Powder X-ray diffraction (XRD) pattern was acquired using X-ray diffractometer with a CuK_α­_ radiation source (SmartLab, Rigaku). The powders with particle sizes less than 45 μm were used for XRD measurements. The powder diffraction pattern was refined with GSAS-II software. XPS was conducted using an in-situ X-ray photoelectron spectrometer (Axis-Supra, Kratos) with a monochromatic Al X-ray source. The binding energies of all the elements were calibrated to the adventitious carbon peak (284.8 eV). The surface contamination was removed by Ar ion cluster gun etching for 60 s. XPS fitting was performed using the least-squares method to avoid any bias from manual fitting. Therefore, the reported spectra consist of a set of deconvoluted Lorentzian peaks that minimize the residual signals between the fitted curves and experimental data. Quantitative analysis of chemical composition was conducted using an inductively coupled plasma-optical emission spectroscopy (ICP, ICP-OES 720, Agilient). Powder samples were completely dissolved in nitric acid using microwave reactor and used for the analysis. The optical reflectance of samples was measured by Fourier transform infrared spectrometer (Nicolet iS50, Thermo Fisher) equipped with Smart Diffuse Reflectance accessories. The signals were recorded from 650 to 4000 cm^−1^ by DLaTGS detector. The reflectance spectra were accumulated 64 times and were converted to Kubelka–Munk function by F(R) = (1 − R)^2^/2R, where R is the reflectance.

The high-resolution synchrotron X-ray diffraction and total scattering measurements were performed at beamline ID31 at the European Synchrotron Radiation Facility (ESRF). The sample powders were loaded into cylindrical slots (approx. 1 mm thickness) held between Kapton windows in a high-throughput sample holder. Each sample was measured in transmission geometry with an incident X-ray energy of 75.051 keV (λ = 0.16520 Å). Measured intensities were collected using a Pilatus CdTe 2M detector (1679 × 1475 pixels, 172×172 µm^2^ each) positioned with the incident beam in the corner of the detector. The sample-to-detector distance was approximately 1.5 m for the high-resolution measurements and 0.3 m for the total scattering measurements. Background measurements for the empty windows were measured and subtracted. NIST SRM 660b (LaB_6_) was used for geometry calibration performed with the software pyFAI followed by image integration including a flat-field, geometry, solid-angle, and polarization corrections. All the sealed sample vials were opened in an argon glove box (O_2_ and H_2_O levels below 0.1 ppm). In the glove-box the samples were loaded into the sample holder. The sample holder was transferred to the beamline ID31 in a closed container. The container was open 10 min. before the measurements and the sample holder kept in a desiccator (2 hours) in between the XRD and TS-PDF measurements.

Active learning framework

In this study, an active learning framework is employed to efficiently explore a complex, multidimensional design space. This approach iteratively queries the experimental system for labeling new data points, enabling the model to rapidly assimilate information.

At the core of our active learning implementation is the Gaussian Process (GP). GP offers a probabilistic approach to learning within kernel-defined function spaces, crucially providing not just predictions but also estimations of uncertainty. The active learning model was initially trained on a set of 22 randomly selected compositions, including several endpoint compositions where fractions of more than two elements are zero to ensure a diverse starting point. As the learning process progressed, each iteration involved the integration of 10 new data points into the training set. The selection of data points for labeling was guided by the Expected Improvement (EI) method. This method quantitatively evaluates the potential contribution of each candidate point to the performance of the model. The EI for a point is defined by the formula:

$$EI\left( x \right)=\int_{-\infty}^{\infty} \max\left( 0, f\left( x \right)-f\left( x^{+} \right) \right)\varphi\left( z \right)dz$$

, where $x$ represents an unlabeled data point. The term $f\left( x \right)$ represents the model’s prediction value for point $x$, while $f\left( x^{+} \right)$ denotes the current best observation. $\varphi\left( z \right)$ is the probability density function of the normal distribution $\mathcal{N}\left( 0, 1 \right)$.

Thermoelectric efficiency estimation

The TE performance of discovered materials was evaluated by calculating the maximum TE efficiency (η_max_). Typically, η_max_ is routinely calculated via the following equation:

$$\eta_{max}=\frac{\Delta T}{T_{h}}\frac{\sqrt{1+Z\cdot T_{avg}}-1}{\sqrt{1+Z\cdot T_{avg}}+\frac{T_{c}}{T_{h}}}$$

, where $T_{h}$, $T_{c}$, $\Delta T$, and $T_{avg}$ are the temperatures of hot side, cold side, temperature difference ($T_{h}-T_{c}$), and average temperature ($\frac{T_{h}+T_{c}}{2})$, respectively ^1^. However, this equation does not accurately give the TE efficiency because the TE properties significantly vary with the temperature, and the TE leg does not show a uniform temperature along its dimension. Therefore, considerations on the temperature distribution of the TE leg were made by solving the following one-dimensional differential equation on TE properties:

$$\frac{d}{dx}\left( \kappa\left( T \right)\frac{dT}{dx} \right)+\frac{1}{\sigma\left( T \right)}\left( \frac{I}{A} \right)^{2}-\left( \frac{dS}{dT} \right)\left( \frac{dT}{dx} \right)T\left( \frac{I}{A} \right)=0$$

, where $I$ is the electrical current and $A$ is the cross-sectional area of the TE leg. We set the boundary conditions as $T\left( x=0 mm \right)=323 K$ and $T\left( x=8 mm \right)=773 K$, and $A$ was set to 9 mm^2^, similar to the values in the real experiments. Then, the TE efficiency is given by the ratio of the generated power to the input heat from the hot side as follows:

$$\eta\left( I \right)=\frac{P\left( I \right)}{Q_{h}\left( I \right)}=\frac{I\left( V_{gen}-IR \right)}{-\kappa\left( T_{h} \right)A\left( \frac{dT}{dx} \right)_{T_{h}}+I\alpha\left( T_{h} \right)T_{h}}$$

, where $V_{gen}$ is the generated output voltage of a TE leg. The temperature-dependent TE parameters were interpolated at using barycentric polynomial interpolation at eleven Chebyshev temperature nodes.

Thermoelectric quality factor calculation

The quality factor *B* is proportional to the ratio of the weighted mobility ($\mu_{w}$) and the lattice thermal conductivity ($\kappa_{latt}$), expressed as:

$$B=\frac{8\pi k_{B}\left( 2m_{0} \right)^{\frac{3}{2}}}{3eh^{3}}{(k_{B}T)}^{5/2}\frac{\mu_{w}}{\kappa_{latt}}$$

where $k_{B}$ is the Boltzmann constant, $m_{0}$ is the electron mass, $e$ is the elementary charge, $h$ is Planck’s constant, and $T$ is the absolute temperature ^2^. If we neglect the effect of the carrier-induced lattice softening (i.e., reduced $\kappa_{latt}$ at high carrier concentration) ^3^, *B* remains independent of carrier concentration while still capturing the influence of scattering mechanism, band structure (via $\mu_{w}$) and phonon scattering (via $\kappa_{latt}$).

**Supplementary Note 1. Effect of temperature averaging on descriptor bias**

The averaging procedure could, in principle, bias the active learning (AL) model toward materials with more uniform *zT* values across the temperature range. While such direction is indeed desirable for thermoelectric power generation efficiency, it could potentially skew the training process. To assess this potential bias, we performed a correlation analysis between temperature and both *B* and *zT*. The results (Fig. S12 and S13) show only a weak linear correlation between *T* and *B* (*r*=0.22) and essentially no correlation between *T* and *zT* (*r*=0.10). These findings indicate that the averaging procedure does not introduce a strong or systematic bias toward uniformly high *zT* profiles.

That said, we acknowledge that more rigorous treatments of temperature dependence could further refine the model. In the current AL framework, where optimization is typically performed with a single-objective function, directly embedding the full temperature dependence of *B* or *zT* remains challenging. Nonetheless, we envision that future extensions—such as multi-objective optimization schemes or advanced descriptors explicitly encoding temperature-dependent behavior—could mitigate any residual bias and further enhance predictive accuracy.

**Supplementary Note 2. Nonparabolicity, multiband, and scattering effect in estimating *B***

The HECs may exhibit highly complicated band structures with significant non-parabolicity. Nevertheless, both theoretical formulation and experimental evidence indicate that *B* remains a valid and effective descriptor for screening high-performance thermoelectric materials.

As described in the manuscript, *B* is mathematically expressed as

$$B=\frac{8\pi k_{B}\left( 2m_{0} \right)^{\frac{3}{2}}}{3eh^{3}}{(k_{B}T)}^{5/2}\frac{\mu_{w}}{\kappa_{latt}}$$

where $k_{B}$ is the Boltzmann constant, $m_{0}$ is the electron mass, $e$ is the elementary charge, $h$ is Planck’s constant, and $T$ is the absolute temperature. Given that $k_{B}$, $m_{0}$, $e$, $h$, and $T$ are the constant, the variables in the formula are $\mu_{w}$ and $\kappa_{latt}$; therefore, we scrutinize whether these parameters are affected by the assumption of non-parabolicity and presence of multibands.

First, $\mu_{w}$ was calculated according to the following equation:^4^

$$\mu_{w}=\frac{3h^{3}\sigma}{8\pi e{(2m_{0}k_{B}T)}^{3/2}}\left[ \frac{exp\left[ \left( \frac{\left| S \right|}{k_{B}/e}-2 \right) \right]}{1+exp\left[ -5\left( \frac{\left| S \right|}{k_{B}/e}-1 \right) \right]}+\frac{\frac{3}{\pi^{2}}\frac{|S|}{k_{B}/e}}{1+exp\left[ 5\left( \frac{\left| S \right|}{k_{B}/e}-1 \right) \right]} \right]$$

where $m_{0}$ is the electron mass, $k_{B}$ is the Boltzmann constant, $e$ is the elementary charge, $h$ is Planck’s constant, and $T$ is the absolute temperature. Here, the calculation of $\mu_{w}$ using the above equation does not assume single parabolic band and acoustic phonon scattering. Therefore, the $\mu_{w}$ values are valid regardless whether they are obtained from single parabolic band materials (e.g., Si), Kane band materials (e.g., CoSb_3_), or Multiband materials (e.g., PbTe).

Then, we discuss the effect of non-parabolicity and multiband effect on the deviation for estimating the lattice thermal conductivity. The lattice thermal conductivity ($\kappa_{l}$) is calculated by subtracting the electronic contribution of the thermal conductivity ($\kappa_{e}$) from the total thermal conductivity ($\kappa_{tot}$) as the following equation:

$$\kappa_{l}=\kappa_{tot}-\kappa_{e}$$

Here, $\kappa_{e}$ is obtained from the Wiedemann-Franz law as follows:

$$\kappa_{e}=L\sigma T$$

,where $L$, $\sigma$, and $T$ is the Lorenz number, electrical conductivity, and absolute temperature, respectively. Conventionally, $L$ was often assumed to be the constant value of 2.44×10^-8^ WΩK^-2^ (limit for degenerate semiconductors). However, Kane band materials with non-parabolicity may exhibit ~40% deviation of $L$ if we assume the degenerate limit. Therefore, the experimentally measured Seebeck coefficient is used for the estimation of $L$ as follows:^5^

$L=1.5+exp\left[ -\frac{|S|}{116} \right]$ (in 10^-8^ WΩK^-2^)

This equation gives relatively accurate value of $L$ (~5% deviation) under the single parabolic band and acoustic phonon scattering assumption. However, it is proven that this equation is still valid even for Kane band, multiple bands, and scattering mechanisms other than acoustic phonon scattering. The estimated error for $L$ in this case is reported to be within 20%; in fact, PbSe-based materials with Kane band and polar optical scattering exhibited maximum error to be 19.5%,which is well within the uncertainty margin of experimental thermoelectric property evaluation and thus the validity of the above equation without assuming single parabolic band and acoustic phonon scattering assumptions.

From the above discussion, we showed that the obtained $B$ value is experimentally and theoretically valid without assuming the single parabolic band and acoustic phonon scattering. This robustness is further corroborated by our experimental dataset: a strong linear correlation (*r*=0.958, *R*^2^=0.918, *p*<10^-30^) is observed between *zT*_avg_ and *B*_avg_ across diverse HEC samples (Fig. S14), confirming that *B* remains statistically significant and practically relevant even for materials without a simple parabolic band structure.


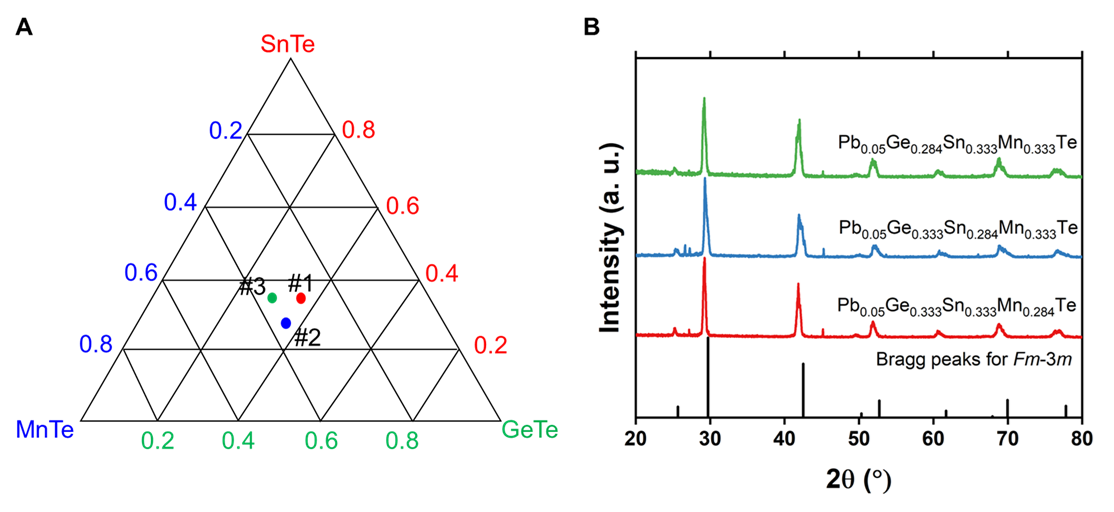
Fig. S1.

Pseudo-ternary diagram of GeTe-MnTe-SnTe with a Pb concentration of 5 at.% (A) and the corresponding powder XRD pattern of the samples, showing secondary phase formation and evidence of phase separation.


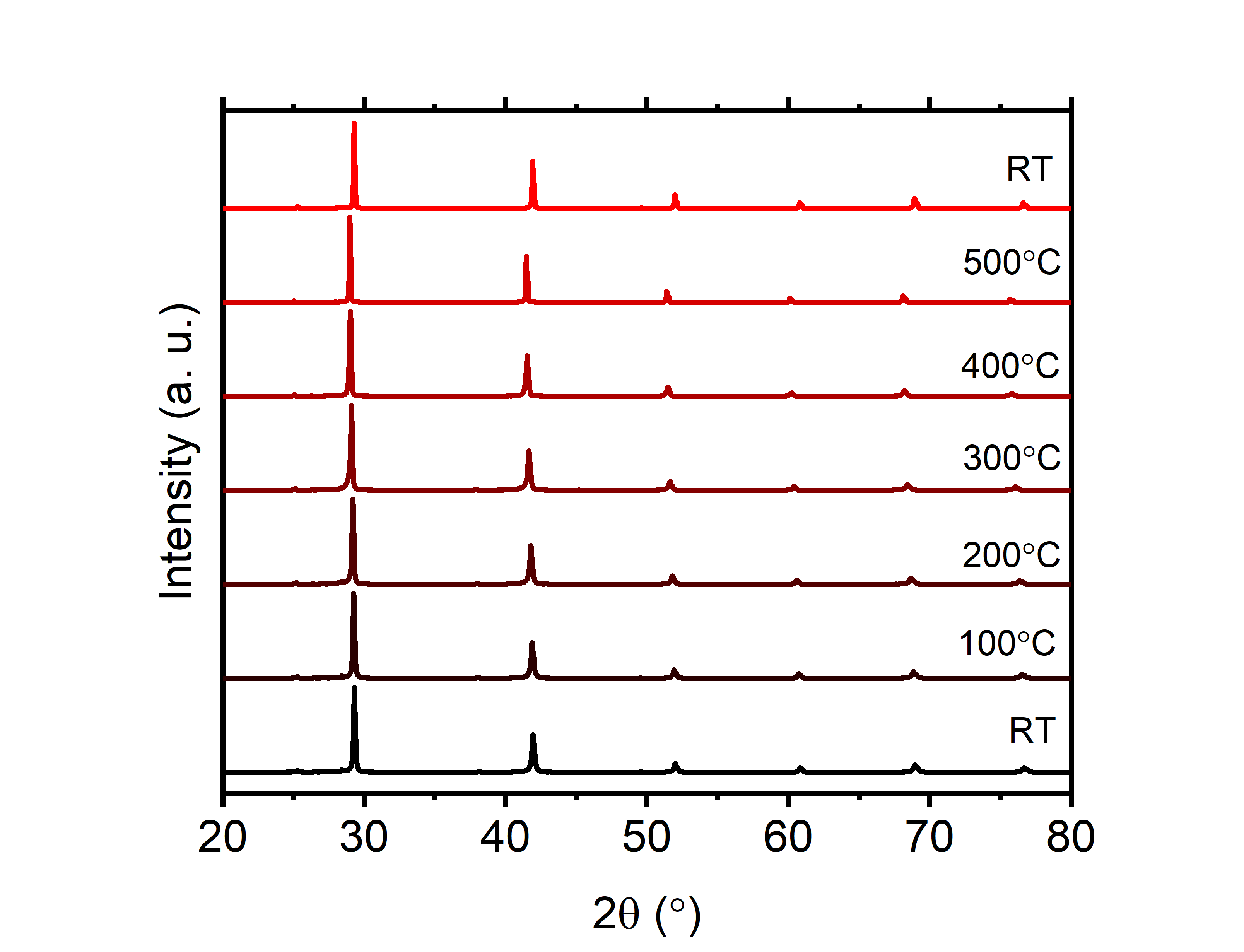


Fig. S2.

High-temperature powder XRD pattern of AL71 from 298 K to 773 K. The XRD pattern after cooling back to 298 K is shown at the top.


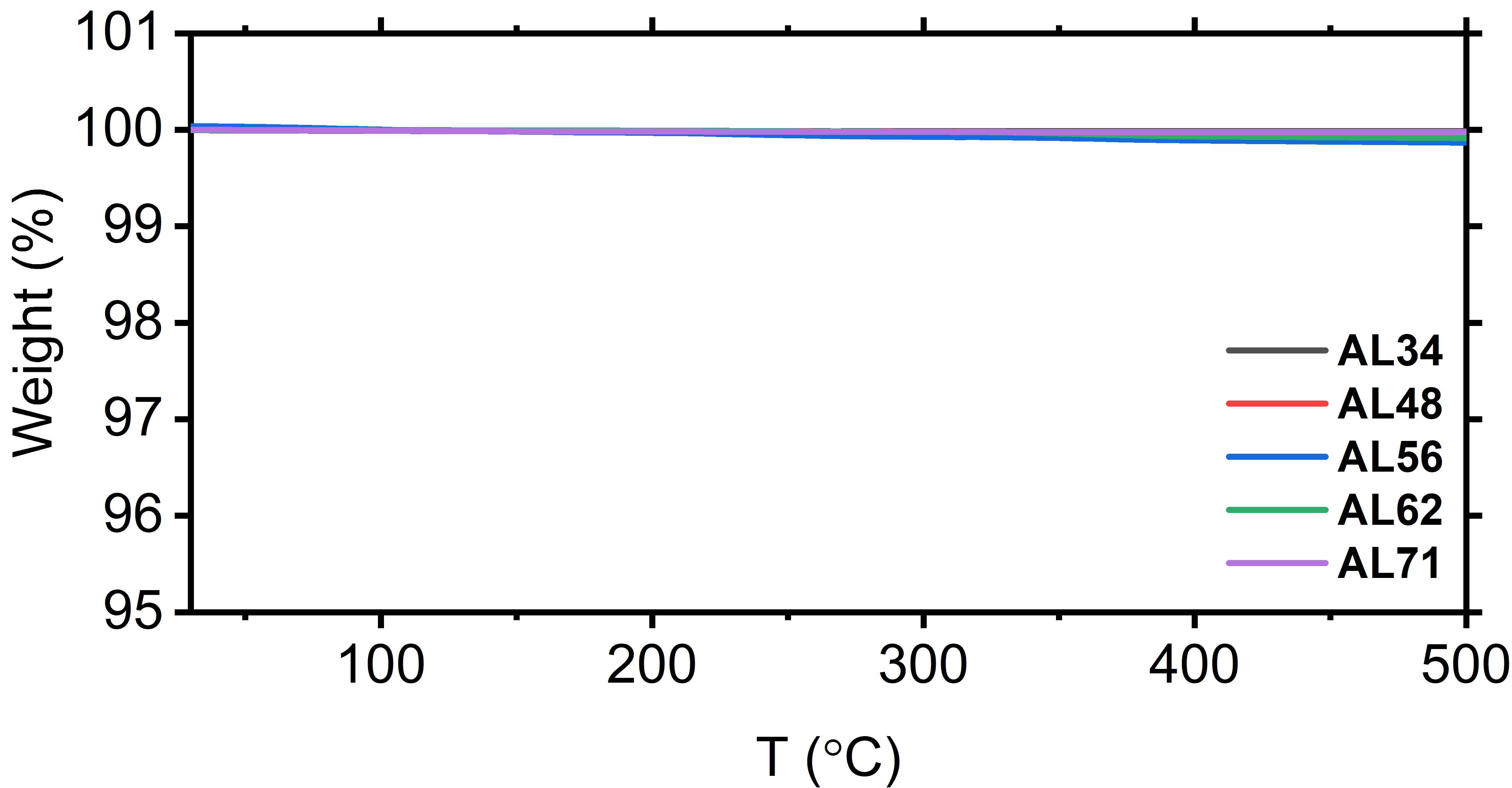


Fig. S3.

Thermogravimetric analysis (TGA) results for five representative samples from each batch, showing negligible mass loss upon heating to 773 K.


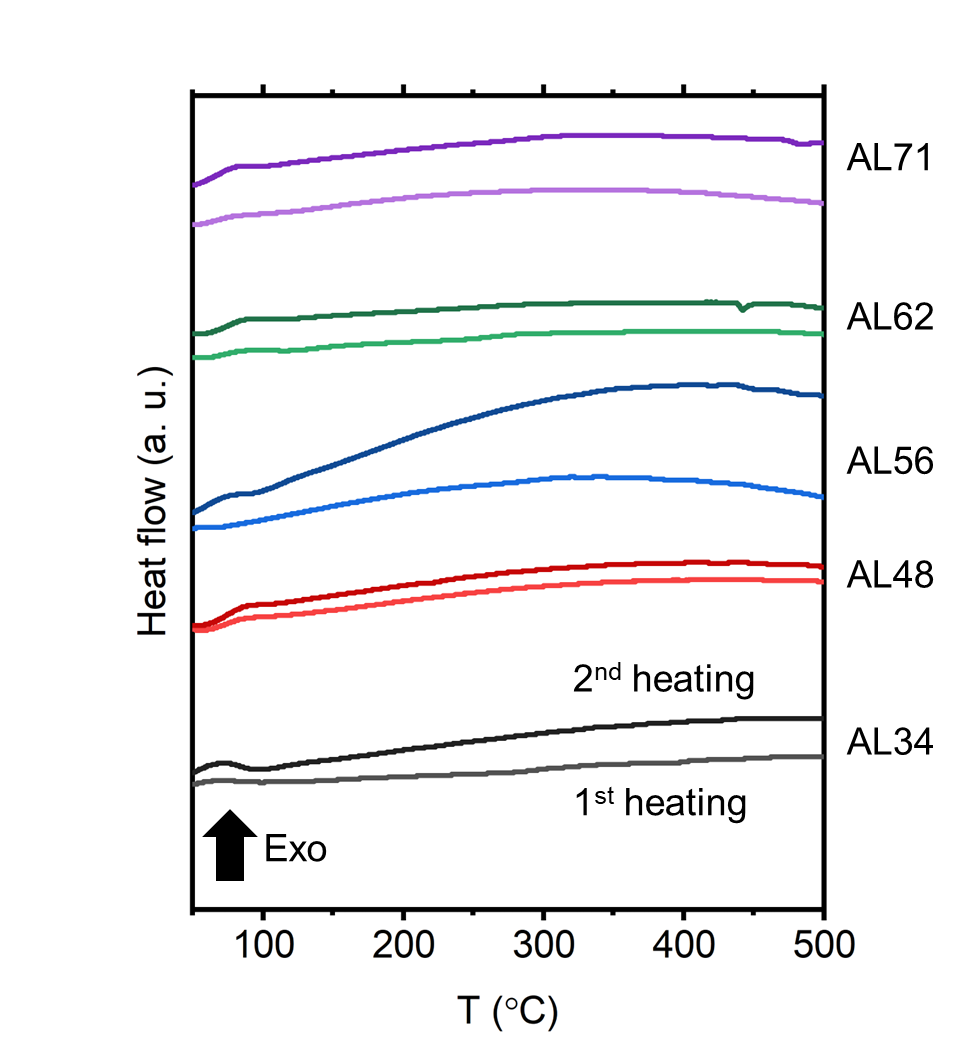


Fig. S4.

Differential scanning calorimetry (DSC) results for five representative samples from each batch. Measurements were conducted over two consecutive heating cycles to confirm the thermal stability of the samples.


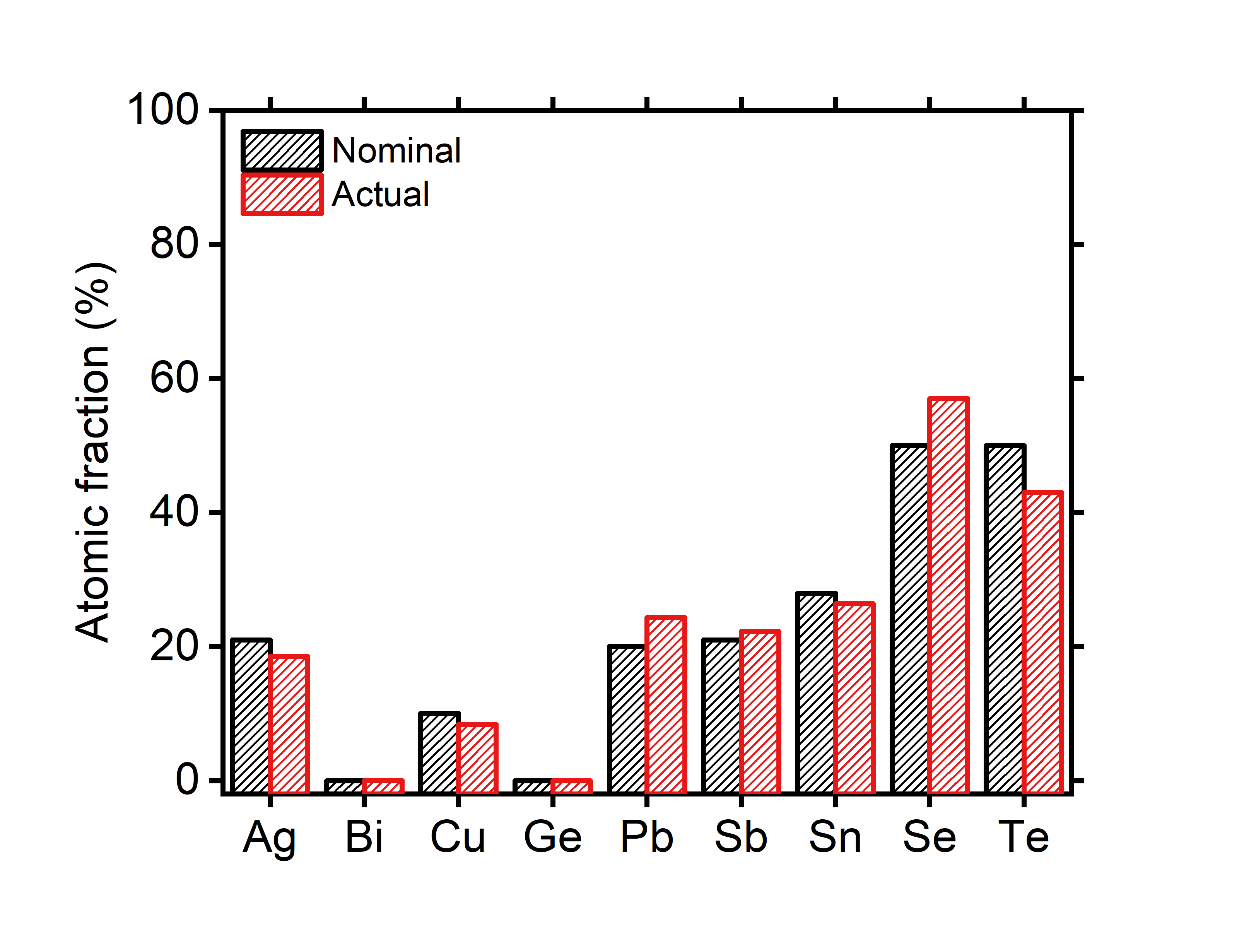


Fig. S5.

ICP-OES elemental quantification results of AL56.


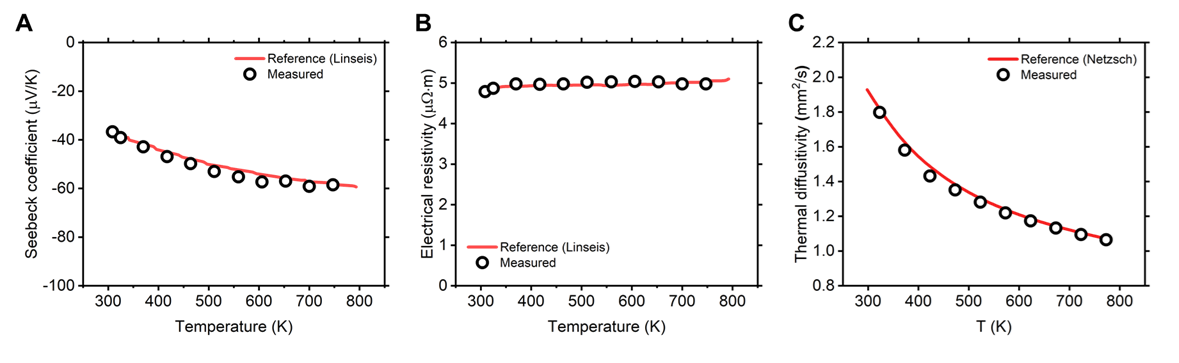


Fig. S6.

Standard material measurement results using a constantan alloy and ZEM-3 for Seebeck coefficient (A) and electrical resistivity (B). Calibration of LFA467 was performed using Pyroceram 9606 as a standard sample, showing excellent agreement of thermal diffusivity (C) with the reference.


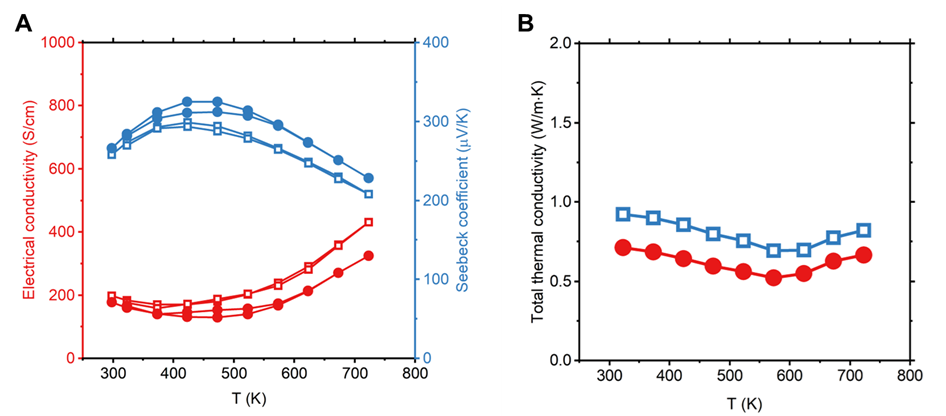


Fig. S7.

Electrical conductivity and Seebeck coefficient of the ALV sample (A) and thermal conductivity of the ALV sample during a heating-cooling cycle. Open and closed symbols denote samples from the first and second batches, respectively.


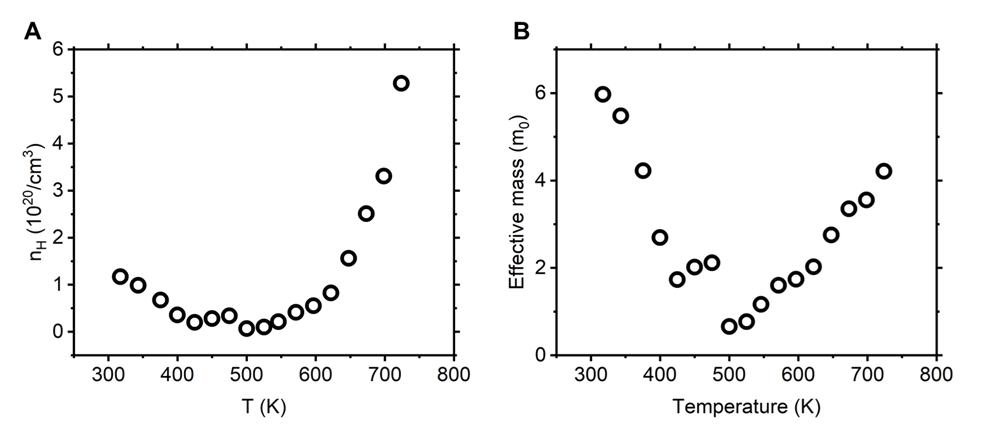


Fig. S8.

High-temperature Hall measurement of the ALV sample, showing the temperature dependence of Hall carrier concentration (A) and the calculated effective mass (B).


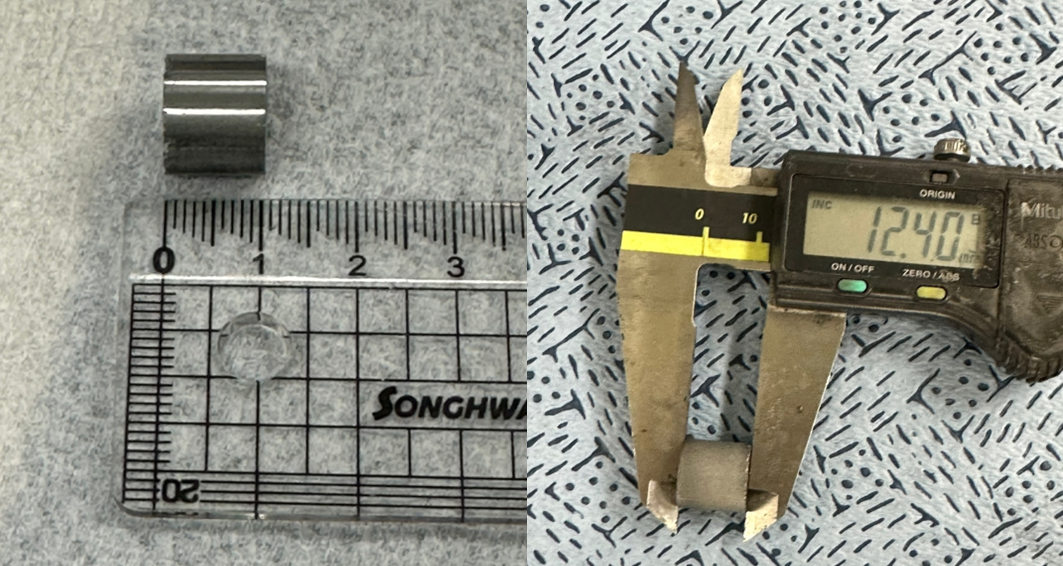


Fig. S9.

Digital photograph of a high-quality polycrystalline HEC sample after the SPS process, showing a metallic luster when polished. All thermoelectric properties were measured along the press direction to minimize potential errors due to sample anisotropy. To ensure consistency in the measurement direction, all sintered pellets were prepared with a thickness greater than 10 mm.


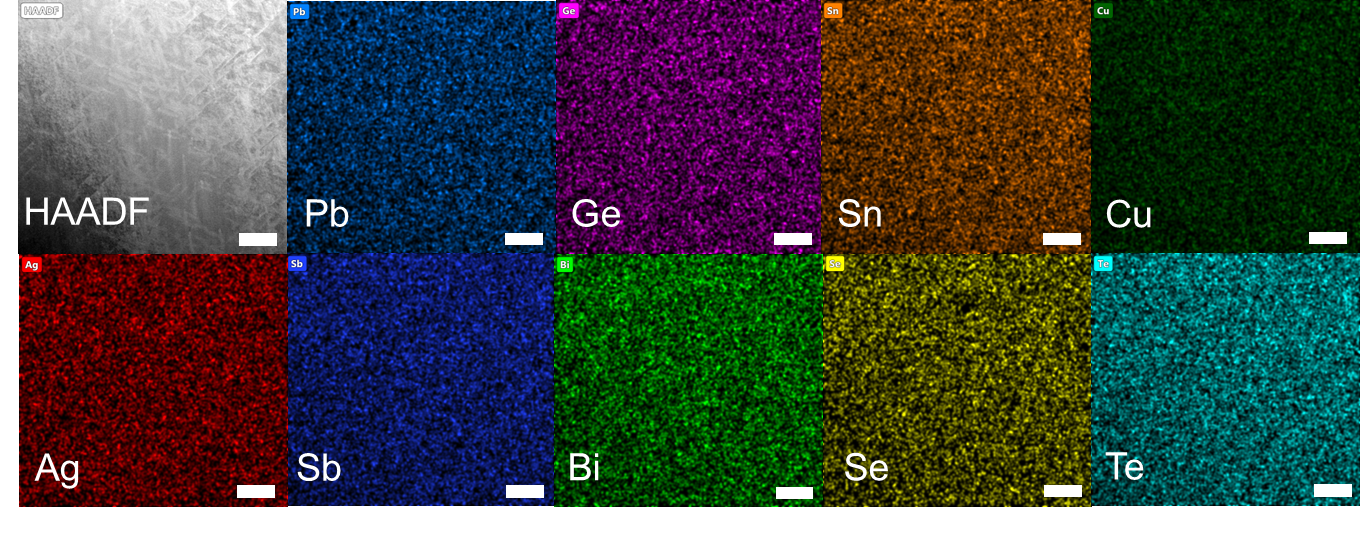


Fig. S10.

Additional STEM-EDS mapping results of the nonary Pb_0.2_Ge_0.13_Sn_0.15_Cu_0.1_Ag_0.21_Bi_0.08_Sb_0.13_Se_0.5_Te_0.5_ alloy at lower magnification. The mapping integration time was 600 s.

*
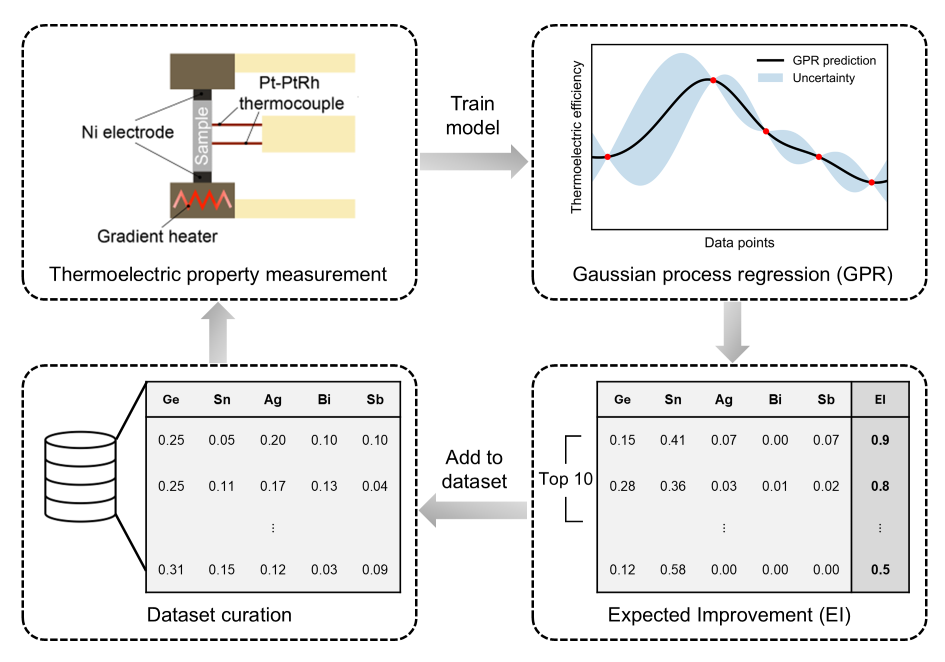
*Fig. S11.

Closed-loop active learning workflow for high-entropy thermoelectric chalcogenide discovery. The iterative optimization cycle consists of four steps: (i) dataset curation; (ii) experimental synthesis and measurement of the temperature-averaged thermoelectric quality factor (Bavg); (iii) Gaussian process regression (GPR) trained on the current dataset to produce predictions (black curve) and posterior uncertainty (blue shading) over the compositional space; and (iv) Expected Improvement (EI) to select the top 10 candidate compositions. The selected candidates are added to the dataset, closing the loop and returning to step (i) for the next cycle.


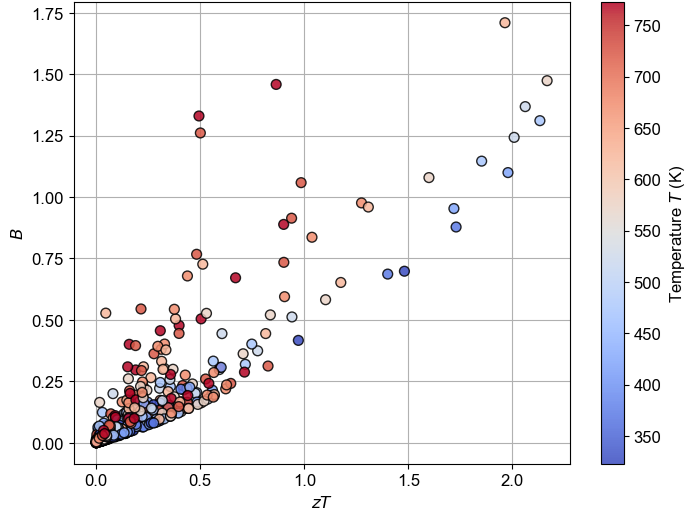


Fig. S12.

Scatter plot showing the distribution of *zT* and *B* with the corresponding temperature.


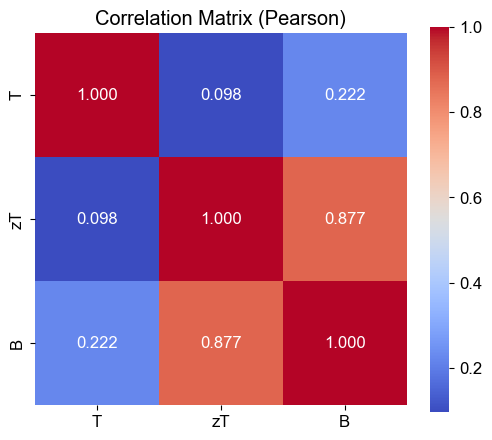


Fig. S13.

Pearson correlation matrix of *T*, *zT*, and *B* of the HEC samples.


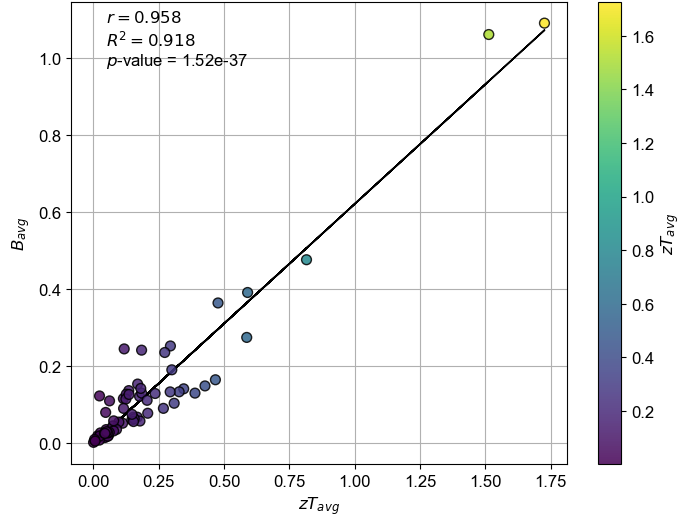


**Fig. S14.**

Scatter plot of experimentally measured *zT*_avg_ and *B*_avg_ for the HEC samples. A strong positive linear correlation is observed (*r*=0.958, *R*^2^=0.918). The correlation is statistically significant (*p*<10^-30^), indicating that the relationship between *zT*_avg_ and *B*_avg_ is unlikely to arise from random variation.


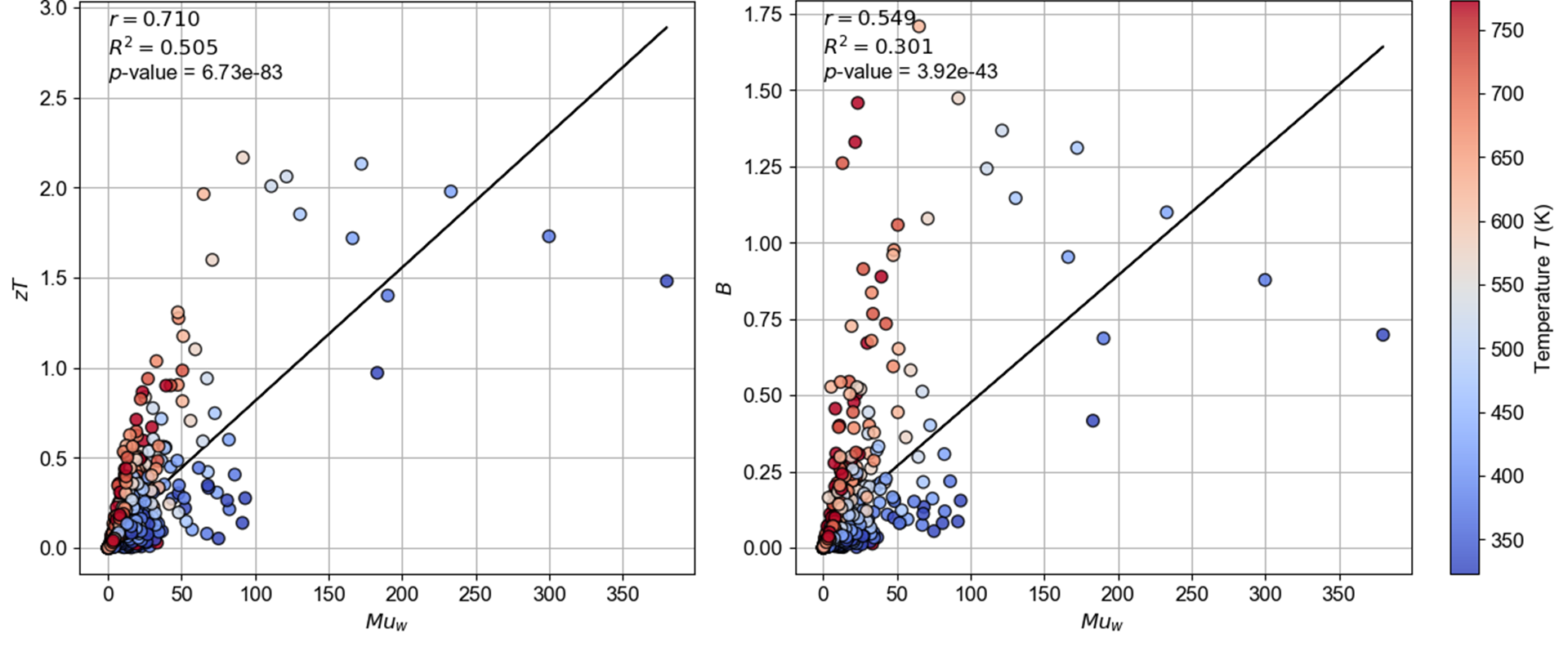


**Fig. S15.**

Scatter plot of the weighted mobility ($\mu_{w}$) and *zT* (left) and *B* (right). Both plots show that $\mu_{w}$ alone does not determine high thermoelectric performance in HECs.

Table S1.

Reported *zT* values of high-entropy thermoelectric alloys shown in Fig. 1E

| Number | Composition | Peak zT | Reported year | References |
| --- | --- | --- | --- | --- |
| 1 | Pb_0.89_Sb_0.012_Sn_0.1_Se_0.5_Te_0.25_S_0.25_ | 1.8 @ 900K | 2021 | ^6^ |
| 2 | Ga_0.025_(Sn_0.25_Pb_0.25_Mn_0.25_Ge_0.25_)_0.975_Te | 1.52 @ 823K | 2021 | ^7^ |
| 3 | (Sn_0.74_Ge_0.2_Pb_0.1_)_0.75_Mn_0.275_Te | 1.42 @ 850K | 2018 | ^8^ |
| 4 | Pb_0.935_Na_0.025_Cd_0.04_Se_0.5_S_0.25_Te_0.25_ | 2.0 @ 900K | 2021 | ^9^ |
| 5 | AgMnSn_0.25_Pb_0.75_SbTe_4_ | 1.3 @ 773K | 2022 | ^10^ |
| 6 | AgMnGePbSbTe5 - 3% Ag2Te | 2.46 @ 750K | 2024 | ^11^ |
| 7 | Ge_0.61_Ag_0.11_Sb_0.13_Pb_0.12_Bi_0.01_Te | 2.7 @ 750K | 2022 | ^12^ |

**References**

1 Kim, H. S., Liu, W., Chen, G., Chu, C.-W. & Ren, Z. Relationship between thermoelectric figure of merit and energy conversion efficiency. *Proc. Natl. Acad. Sci. U. S. A.* **112**, 8205-8210 (2015). <https://doi.org:doi:10.1073/pnas.1510231112>

2 Wang, H., Pei, Y., LaLonde, A. D. & Jeffery Snyder, G. Material design considerations based on thermoelectric quality factor. *Thermoelectric Nanomaterials: Materials Design and Applications*, 3-32 (2013).

3 Slade, T. J. *et al.* Charge-carrier-mediated lattice softening contributes to high zT in thermoelectric semiconductors. *Joule* **5**, 1168-1182 (2021).

4 Snyder, G. J. *et al.* Weighted mobility. *Adv. Mater.* **32**, 2001537 (2020).

5 Kim, H. S., Gibbs, Z. M., Tang, Y. L., Wang, H. & Snyder, G. J. Characterization of Lorenz number with Seebeck coefficient measurement. *APL Mater.* **3** (2015). <https://doi.org:Artn> 041506

10.1063/1.4908244

6 Jiang, B. *et al.* High-entropy-stabilized chalcogenides with high thermoelectric performance. *Science* **371**, 830-834 (2021). <https://doi.org:doi:10.1126/science.abe1292>

7 Wang, X. *et al.* Enhanced Thermoelectric Performance in High Entropy Alloys Sn0.25Pb0.25Mn0.25Ge0.25Te. *ACS Applied Materials & Interfaces* **13**, 18638-18647 (2021). <https://doi.org:10.1021/acsami.1c00221>

8 Hu, L. *et al.* Entropy Engineering of SnTe: Multi-Principal-Element Alloying Leading to Ultralow Lattice Thermal Conductivity and State-of-the-Art Thermoelectric Performance. *Advanced Energy Materials* **8**, 1802116 (2018). <https://doi.org:https://doi.org/10.1002/aenm.201802116>

9 Jiang, B. *et al.* Entropy engineering promotes thermoelectric performance in p-type chalcogenides. *Nature Communications* **12**, 3234 (2021). <https://doi.org:10.1038/s41467-021-23569-z>

10 Ma, Z. *et al.* High Thermoelectric Performance and Low Lattice Thermal Conductivity in Lattice-Distorted High-Entropy Semiconductors AgMnSn1–xPbxSbTe4. *Chemistry of Materials* **34**, 8959-8967 (2022). <https://doi.org:10.1021/acs.chemmater.2c02344>

11 Ma, Z. *et al.* Synergistic Performance of Thermoelectric and Mechanical in Nanotwinned High-Entropy Semiconductors AgMnGePbSbTe5. *Advanced Materials* **36**, 2407982 (2024). <https://doi.org:https://doi.org/10.1002/adma.202407982>

12 Jiang, B. *et al.* High figure-of-merit and power generation in high-entropy GeTe-based thermoelectrics. *Science* **377**, 208-213 (2022).
